# Transcriptomic analysis of the m^6^A reader *YTHDF2* in the maintenance and differentiation of human embryonic stem cells

**DOI:** 10.1093/stmcls/sxaf032

**Published:** 2025-05-26

**Authors:** Boshi Feng, Yanxi Chen, Huanchang Tu, Jin Zhang, Lingling Tong, Xiaohan Lyu, Aaron Trent Irving, Di Chen

**Affiliations:** Center for Reproductive Medicine of The Second Affiliated Hospital, Center for Regeneration and Cell Therapy of Zhejiang University-University of Edinburgh Institute (ZJU-UoE Institute), Zhejiang University School of Medicine, Zhejiang University, Hangzhou, Zhejiang 310003, China; Center for Reproductive Medicine of The Second Affiliated Hospital, Center for Regeneration and Cell Therapy of Zhejiang University-University of Edinburgh Institute (ZJU-UoE Institute), Zhejiang University School of Medicine, Zhejiang University, Hangzhou, Zhejiang 310003, China; Center for Reproductive Medicine of The Second Affiliated Hospital, Center for Regeneration and Cell Therapy of Zhejiang University-University of Edinburgh Institute (ZJU-UoE Institute), Zhejiang University School of Medicine, Zhejiang University, Hangzhou, Zhejiang 310003, China; Center for Reproductive Medicine of The Second Affiliated Hospital, Center for Regeneration and Cell Therapy of Zhejiang University-University of Edinburgh Institute (ZJU-UoE Institute), Zhejiang University School of Medicine, Zhejiang University, Hangzhou, Zhejiang 310003, China; Center for Reproductive Medicine of The Second Affiliated Hospital, Center for Regeneration and Cell Therapy of Zhejiang University-University of Edinburgh Institute (ZJU-UoE Institute), Zhejiang University School of Medicine, Zhejiang University, Hangzhou, Zhejiang 310003, China; Centre of Infection Immunity and Cancer (IIC) of Zhejiang University-University of Edinburgh Institute (ZJU-UoE Institute), Zhejiang University School of Medicine, Zhejiang University, Hangzhou, Zhejiang 310029, China; Centre of Infection Immunity and Cancer (IIC) of Zhejiang University-University of Edinburgh Institute (ZJU-UoE Institute), Zhejiang University School of Medicine, Zhejiang University, Hangzhou, Zhejiang 310029, China; Edinburgh Medical School: Biomedical Sciences, College of Medicine and Veterinary Medicine, The University of Edinburgh, Edinburgh EH8 9AG, United Kingdom; Center for Reproductive Medicine of The Second Affiliated Hospital, Center for Regeneration and Cell Therapy of Zhejiang University-University of Edinburgh Institute (ZJU-UoE Institute), Zhejiang University School of Medicine, Zhejiang University, Hangzhou, Zhejiang 310003, China; Edinburgh Medical School: Biomedical Sciences, College of Medicine and Veterinary Medicine, The University of Edinburgh, Edinburgh EH8 9AG, United Kingdom; State Key Laboratory of Biobased Transportation Fuel Technology, Haining, Zhejiang 314400, China

**Keywords:** hESCs, neural development, *ROBO1*, *YTHDF2*

## Abstract

As the most abundant internal modification on mRNAs, *N*^6^-methyladenosine (m^6^A) has been discovered to be involved in different biological processes. Mostly determined by m^6^A methyl-transferases (m^6^A writers) and demethylases (m^6^A erasers), different cell types possess differential m^6^A profiles of transcriptomes. However, the interpretation of the m^6^A-encoded epitranscriptomic information needs m^6^A readers to bind and recruit different machinery for regulating the target mRNAs, which in turn, may regulate cell fates. The functions of the m^6^A readers in the regulation of the maintenance and differentiation of human embryonic stem cells (hESCs) remain largely unknown. In this study, we deleted the whole genomic region of the m^6^A reader *YTHDF2* and discovered that *YTHDF2* is dispensable for the maintenance, but important for the differentiation of hESCs, especially for the differentiation towards ectoderm. Furthermore, we identified the m^6^A-modified *ROBO1* mRNAs as potential targets by YTHDF2 in regulating hESC to neuroectoderm differentiation. This study reveals the potential roles of the m^6^A reader *YTHDF2* in regulating the specification of neuroectodermal cell fate.

## Graphical Abstract

**Graphical Abstract:**
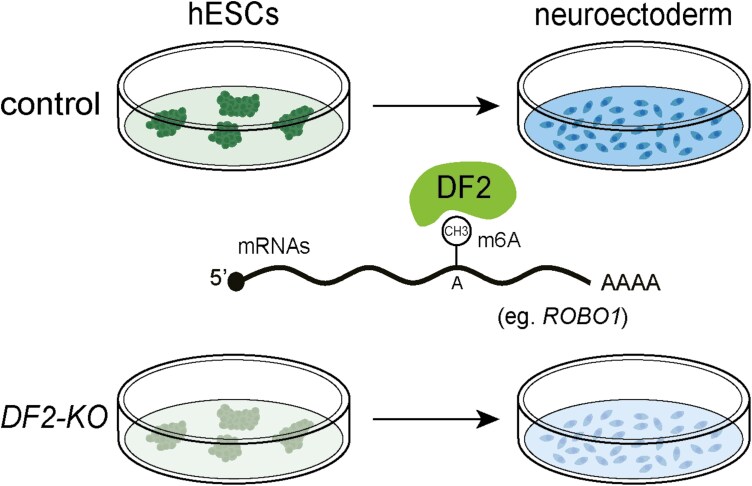

**Significance Statement**

Human embryonic stem cells hold the promise for regenerative medicine by the potential to differentiate into most of the functional cell types in human bodies. Understanding the precise regulatory machinery governing the differentiation of human embryonic stem cells is the key for generating pure and functional cell types for clinical applications. In this study, we uncover the regulatory roles of *YTHDF2*, the RNA-binding protein for recognizing m^6^A modification in mRNAs, in the differentiation of neuroectodermal lineage, shedding new light on the m^6^A-mediated epitranscriptomic coordination in the differentiation of human embryonic stem cells.

## Introduction

Embryonic stem cells (ESCs), derived from the inner cell mass (ICM) of the developing blastocysts, are pluripotent cells with the capability of self-renewal and differentiation to produce all cell types in adult organisms.^1^ Mouse and human ESCs (mESCs and hESCs), capturing most of the molecular characteristics of early embryogenesis in mice and humans, respectively, are considered powerful in vitro systems for studying early mammalian development and regulatory mechanisms.^2^ Recent studies have indicated that epigenetic mechanisms, such as DNA modifications, RNA modifications, and histone modifications, can regulate self-renewal, pluripotency, and lineage specification.^3^ However, the specific mechanisms of epigenetic regulation at the RNA level in ESCs and early mammalian development are not fully addressed.

*N* ^6^-methyladenosine (m^6^A), the post-transcriptional RNA epigenetic modification, is the most common and reversible internal modification in the mRNAs of mammals. The dynamic regulation of m^6^A sites can be mediated through the deposition by methyl-transferases (m^6^A writers, including Methyltransferase 3 [*METTL3*], Methyltransferase 14 [*METTL14*], and WT1-associated protein [*WTAP*]) and the removal by demethylases (m^6^A erasers, including FTO alpha-ketoglutarate-dependent dioxygenase [*FTO*] and alkB homolog 5, RNA demethylase [*ALKBH5*]). Furthermore, when m^6^A-modified RNAs are bound by specific RNA-binding proteins (m^6^A readers, comprising members of YTH *N*^6^-methyladenosine RNA-binding protein family [*YTH*], and Insulin-like growth factor 2 mRNA-binding protein [*IGF2BP*] families), a multitude of downstream effects get triggered, such as RNA splicing,^4^ nuclear export,^5^ stability,^6^ and translation.^7^

The reversible and dynamic nature of m^6^A modification in mRNAs and the ability to map the modification profiles across the transcriptomes suggest its important regulatory roles in a variety of biological processes. Recent studies have clarified the role of m^6^A modification in ESCs and early embryonic development. The inactivation of the writer *Mettl3* or *Methyltransferase 16* (*Mettl16*) in mice directly results in embryonic lethality.^8,9^ The *Ythdf2* loss of function in zebrafish has been shown to disrupt the highly regulated maternal-to-zygotic transition, thereby halting embryonic development.^10^ In mice, *Ythdf2*-dependent clearance of m^6^A-modified mRNAs also leads to embryonic lethality, with surviving young mice exhibiting neural developmental dysregulation and female-specific infertility.^11,12^ The disruption of *Mettl3* and *Alkbh5* impairs spermatogenesis, leading to male infertility eventually.^13,14^ Conditional knockout of the nuclear-localized *Ythdc1* leads to alternative splicing defects through changes in alternative polyadenylation and the length of 3′ untranslated region (3′UTR), causing early developmental challenges in oocytes.^4,15^*YTHDC2* is also crucial for the normal progression of meiosis and fertility, functioning in promoting RNA degradation and effective translation.^16-18^ Therefore, m^6^A modification in mRNAs is essential for the regulation of gametogenesis and embryogenesis.

Although the profiles of m^6^A-modified mRNAs are determined by m^6^A writers and erasers, how m^6^A modification affects mRNA metabolism, which in turn regulates cell fate, is largely determined by the m^6^A readers and their interacting proteins. However, much less is known about how m^6^A readers are involved in the maintenance and differentiation of hESCs. In this study, we generated the knock-out lines of *YTHDF2* in hESCs. By carefully assessing the maintenance and differentiation of *YTHDF2* knock-out hESCs, we discovered the potential roles of *YTHDF2* in the differentiation of hESCs towards neural cell fate.

## Materials and methods

### Plasmid construction

To generate *YTHDF2-KO* hESC lines, vector plasmids named PL552 (Addgene #68407) with HSV-TK sequences added were used to construct knock-in donor plasmids, while pSpCas9 (BB)-2A-Puro (PX459) V2.0 (Addgene #62988) was used to construct gRNAs and Cas9 expression plasmids. Two 1000 bp fragments, which are before the *YTHDF2* initiation codon and after termination codon separately, were obtained using Phanta Max Super-Fidelity DNA Polymerase (Vazyme, P505-d3) with hESC genome as template and inserted into 2 PL552s with puromycin or blasticidin antibiotic resistance separately.

For the PX459s, Guide RNAs (gRNAs) were designed using CHOPCHOP (http://chopchop.cbu.uib.no). gRNA sequences with sticky ends were synthesized (Beijing Tsingke Biotech Co., Ltd.) and inserted into the PX459 plasmid. The gRNAs used for *YTHDF2-KO* are listed below:

**Table:** 

sgRNA 1	sgRNA 2
5′-ACGAGGTTTCGACTAACACC-3′	5′-TTACCTCTGTA GGAACGTCA-3′

To generate YTHDF2-ADAR- and YTHDF2-mCherry-overexpressing hESC lines, the tetON-YTHDF2-ADAR and tetON-YTHDF2-mCherry plasmids were constructed from the FUW-tetO-loxP-hNANOG (Addgene #68409) vector plasmid. Specifically, YTHDF2 and ADAR full-length coding sequences (CDS) were amplified from human cDNA, and *mCherry* was amplified from lenti U6-sgRNA/EF1a-mCherry (Addgene #114199) using Phanta Max Super-Fidelity DNA Polymerase (Vazyme, P505-d3) and inserted into FUW-tetO-loxP-hNANOG.

To generate dCas13b-ALKBH5-crRNA-ROBO1 hESC lines, the tetON-dCas13b-ALKBH5 and pLenti-U6-crRNA-ROBO1-EF1a-mCherry plasmids were constructed. For the tetON-dCas13b-ALKBH5 plasmids, CDS of *ALKBH5* was amplified with Phanta Max Super-Fidelity DNA Polymerase and replace hNANOG on FUW-tetO-loxP-hNANOG. Then dCas13b sequence was amplified from the dCas13b-FTO plasmid, which is a gift from Shaorong Gao Lab. In order to construct pLenti-U6-crRNA-ROBO1-EF1a-mCherry plasmid, a 36 nt direct repeat (DR sequence) was fused into the pLenti-U6-EF1a-mCherry vector, which is a gift from Chaochen Wang Lab. Then, the crRNA targeting the m^6^A peak on *ROBO1* was designed with CHOPCHOP and cloned into the pLenti-U6-EF1a-mCherry vector with a DR sequence with T4 DNA Ligase (NEB, M0202L). The sequence of DR and crRNA were: CTTTCCACCCAGGGATATGGAGCTTCAA and CACCGTGGATTTGTTCGCATGTCAACAACCTG.

### hESC culture

Normally karyotyped, mycoplasma-free H1 hESCs (passages 40-70) were plated on Matrigel (Corning, 354234)-coated 6-well plate and cultured in hESC Medium (Nuwacell ncTarget hPSC Medium, RP01020) with 50 ng/mL primocin (InvivoGen, ant-pm-2) at 37 °C in 5% CO_2_ incubator. The medium was refreshed once every 2 days. The cells were passaged every 5-6 days using Dispase (STEMCELL, 07923). hESCs were frozen down in freezing media (90% fetal bovine serum [FBS] with 10% dimethyl sulfoxide [DMSO]) in liquid nitrogen and thawed by washing the cells with hESC media before culturing at 37 °C in the 5% CO_2_ incubator.

### 293T culture

293T were plated on 6-cm^2^ plate and cultured in 293T Medium (DMEM high glucose [TransGen Biotech, FI101-01] with 10% FBS [ExcelBio, FSP500], 1× Penicillin-Streptomycin-Glutamine [Thermo Fisher, 10378016], 1% sodium pyruvate [GIBCO, 11360070], and Primocin [InvivoGen, ant-pm-2]) at 37 °C in 5% CO_2_ incubator. The medium was refreshed once every 2 days. The cells were passaged every 5-6 days using 0.25% Trypsin-EDTA (GIBCO, 25300062).

### Cell line construction

To construct *YTHDF2-KO* hESC line, electrotransfection was used to deliver 2 PL552 plasmids and 2 PX459 plasmids (4 μg each) to the cells. P3 Primary Cell 4D-NucleofectorTM X Kit L (Lonza, V4XP-3024) was used to conduct electrotransfection. After transfection, cells were plated on Matrigel-coated 24-well plate in + Y27632 hESC Medium (hESC Medium with 10 μM Y27632 [Selleck, S1049]). Two days after nucleofection, cells were dissociated with Accutase (STEMCELL, 07992) and seeded onto Matrigel-coated 6-well plates for 5 days, when Selection Medium (1 μg/mL Puromycin [Selleck, S7417], 4 μg/mL Blasticidin [Solarbio, B9300], 2 μM/mL Ganciclovir [MCE, 82410-32-0] in + Y27632 hESC Medium) was used to apply antibiotic selection. Then, hESCs were passaged on Matrigel-coated 10-cm^2^ dish at the density of 1.0 × 10^4^ cells/dish for single colony picking. The medium without Y27632 (Selleck, S1049) was changed every 3-4 days until the single colonies grew up. Ninety-six individual colonies were picked and used as template for genomic PCR with 2× Rapid Taq Master Mix (Vazyme, P222-03) on *YTHDF2*. All the experiments were performed for passages 50-70. Primers used in genotyping of *YTHDF2* are listed below:

**Table:** 

Primer names	Sequences
Primer1	5′-GCCTGACCTTACTCATTCCTACC-3′
Primer2	5′-CAGCGGGGCTGCTAAAGCGCATGC-3′
Primer3	5′-CCATCAGAAGCTGGTCGAGA-3′
Primer4	5′-GGATGCCCAAAACCTTCACA-3′
Primer5	5′-GGCTCTACTTCCTCTGCATC-3′

For the YTHDF2-ADAR, YTHDF2-mCherry, and dCas13b-ALKBH5-crRNA-ROBO1 cell lines, lentivirus was used to deliver the tetON-YTHDF2-ADAR, tetON-YTHDF2-mCherry, tetON-dCas13b-ALKBH5, and pLenti-U6-dCas13b-crRNA plasmids to hESCs. At first, 293T cells were dissociated with 0.25% Trypsin and plated on 6-well plate at a density of 5.0 × 10^5^ cells/well in 293T Medium. At about 90% confluency, transfection with 3.2 μg target plasmid, 2.3 μg psPAX2 (Addgene #12260), and 0.8 μg PMD2.G (Addgene #12259) using PEI buffer (Polysciences, 24765100) was conducted. Seventy-two hours after transfection, medium was collected, filtered with 0.45-μm syringe filters (Millipore, SLHU033RB), and concentrated with 10% PEG 8000 (Solarbio, P8260). The lentivirus solution was rested at 4 °C for 24 hours and centrifugated (4 °C, 4000*g*, 30 minutes). After discarding the supernatant, the lentivirus precipitate was resuspended in 50 μL 1× phosphate-buffered saline (PBS) buffer and stored at −80 °C.

To transduce hESCs with lentivirus, hESCs were digested with Accutase and centrifugated. Cell sedimentation was resuspended with 1 mL + Y27632 Medium with 50 μL lentivirus solution and 1 μg/mL Hexadimethrine (Sigma, H9268). Cell-virus mixture was rotated for 2 hours at room temperature. After removal of the virus by centrifugation, hESCs were seeded onto Matrigel-coated 6-well plates.

### Endoderm, mesoderm, and ectoderm induction

To induce endoderm and mesoderm differentiation, hESCs were initially dissociated into single cells using Accutase and seeded onto Matrigel-coated 6-well plates at a density of 5.0 × 10^5^ cells/well, in + Y27632 hESC Medium on day 0. The medium was changed into induction medium and replaced daily from day 1 to day 4: STEMdiff Definitive Endoderm Medium (STEMCELL, 05110) was employed for endoderm induction, while STEMdiff Definitive Mesoderm Medium (STEMCELL, 05220) was employed for mesoderm induction.

To induce ectoderm differentiation, hESCs were dissociated into single cells using Accutase and were seeded onto Matrigel-coated 6-well plates, with STEMdiff Neural induction medium supplemented with SMADi (STEMCELL, 8581) and 10 μM Y27632 on day 0. Y27632 was removed on day 1, and from day 1 to day 6, the medium was changed every day. On day 7, cells were passaged in single-cell form with Accutase, seeded onto Matrigel-coated 6-well plates, and cultured in STEMdiff Neural induction medium supplemented with SMADi (STEMCELL, 8581) for another 6 days, with a density of 2.0 × 10^6^ cells/well.

### Neural progenitor cell induction

hESCs were dissociated into single cells with Accutase and plated in a Matrigel-coated 12-well plate at the density of 7 × 10^4^ cells/well in + Y27632 hESC Medium. The + Y27632 hESC Medium was changed every day until the cells reach 95% confluence, then the neural progenitor cell (NPC) induction medium was changed (50% DMEM/F12 [Gibco, C11330500BT] and 50% Neurobasal medium [Gibco, 21103049] with 1× GlutaMAX [Gibco, 35050061], 0.5× NEAA [Gibco, 11140050], 0.5× N2 [Gibco, 17502048], 5 µg/mL insulin [Beyotime, #P3376-100IU], 100 µM 2‐Mercaptoethanol [Gibco, 21985023], 0.5× B27 [Gibco, 17504044], 100 nM LDN-193189 [Stemgent, 04-0074], 10 μM SB431542 [MCE # HY-10431]). The NPC induction medium was changed once a day for 12 days.

### Immunofluorescence

Cells were washed with 1× PBS buffer after the removal of culture medium, and then were fixed with 4% paraformaldehyde (Biosharp, BL539A) for 10 minutes at room temperature. Cells were permeabilized with 0.1% Triton X-100 in PBS buffer for 15 minutes at room temperature followed by overnight incubation in diluted primary antibodies (anti-OCT3/OCT4 [BD Biosciences, 611203]; anti-YTHDF2 [Proteintech, 24744-1-AP]; anti-SOX1 [CST, 4194S]; anti-TBXT [HUABIO, EM1709-12]; anti-SOX17 [Neuromics, GT15094]; anti-PAX6 [Abcam, ab78545]; anti-FOXG1 [Oasis Biofarm,OB-PGP082]) overnight at 4 °C. After being washed for 3 times with PBS, the cells were incubated with secondary antibodies (Donkey-anti-Mouse [Alexa Fluor 488] [Invitrogen, A21202]; Donkey-anti-rabbit [Alexa Fluor 647] [Abcam,ab150075]; Donkey-anti-goat [Alexa Fluor 647] [Abcam,ab150107]) in dark for 2 hours at room temperature. Then, the samples were stained with DAPI (Beyotime, C1002, 1:5000) in 1% bovine serum albumin (BSA) buffer for 15 minutes. After washing with PBS, cells were mounted on dishes using an anti-fade mounting medium and imaged by Zeiss LSM880 microscope. All images were processed using Image J.

### Western blot

Cells were lysed in 1× sodium dodecyl sulfate-polyacrylamide gel electrophoresis (SDS-PAGE) loading buffer (40% glycerol, 8% SDS, 0.008% bromophenol blue, 5% β-mercaptoethanol, and 0.25 M Tris-HCl, pH 6.8) and boiled at 95 °C for 15 minutes to denature proteins. Then the lysate was loaded onto a 10% Bis-Tris gel, electrophoresed at 120 V for 55 minutes, and transferred onto a PVDF membrane (Millipore, IPVH00010), followed by a blocking with 5% milk in 1× TBST buffer for 30 minutes at room temperature. After that, the PVDF membrane was incubated at 4 °C overnight with diluted primary antibodies (anti-β ACTIN [HUABIO, EM21002, 200 μL]; anti-YTHDF2 [Proteintech, 24744-1-AP, 50 μL]; anti-V5 [sigma, V8012, 50 μg]). The PVDF membrane was washed 3 times with 1× TBST and then incubated with horseradish peroxidase (HRP)-conjugated secondary antibodies (HRP-linked Anti-rabbit IgG [CST, 7074s]; HRP-linked Anti-mouse IgG [CST, 7076s]) for 1 hour at room temperature. After washing 3 more times with 1× TBST, the PVDF membrane was developed using the Western-Bright ECL KIT (Advansta, K12045D50) on an Odyssey Fc Imaging System (LI-COR).

### RT-qPCR

Total RNA was extracted from cells using AG RNAex Pro Reagent (Accurate Biology, AG21101), following the manufacturer’s protocol. Then, total RNA was reverse transcribed using 5× Evo M-MLV RT Master Mix (Accurate Biology, RR037A). Subsequently, cDNAs were mixed with gene-specific primers and 2× SYBR Green Pro Taq HS Premix (Accurate Biology, AG11701) followed by assay on CFX96 Touch Real-Time PCR Detection System (Bio-Rad). Each sample had 2 replicates, and the expression levels were normalized to *GAPDH*. Relative gene expression was calculated using the ΔΔ*C*_*t*_ method. The sequence of primers is listed below:

**Table:** 

Primer names	Sequences
*GAPDH*-F	5′-CGCTTCGCTCTCTGCTCCTCCTGT-3′
*GAPDH*-R	5′-GGTGACCAGGCGCCCAATACGA-3′
*YTHDF2*-F	5′-AGCCCCACTTCCTACCAGATG-3′
*YTHDF2*-R	5′-TGAGAACTGTTATTTCCCCATGC-3′
*NANOG*-F	5′-AGAGGTCTCGTATTTGCTGCAT-3′
*NANOG*-R	5′-AAACACTCGGTGAAATCAGGGT-3′
*OCT4*-F	5′-GCTGGAGCAAAACCCGGAGG-3′
*OCT4*-R	5′-TCGGCCTGTGTATATCCCAGGGTG-3′
*SOX2*-F	5′-TGAATCAGTCTGCCGAGAATCC-3′
*SOX2*-R	5′-TCTCAAACTGTGCATAATGGAGT-3′
*FOXA2*-F	5′-ACCCGGTTTTATCCCTTGAATC-3′
*FOXA2*-R	5′-ATACAACCTGCAACCAGACAGG-3′
*SOX17*-F	5′-TTCGTGTGCAAGCCTGAGAT-3′
*SOX17*-R	5′-TAATATACCGCGGAGCTGGC-3′
*TBXT*-F	5′-AGCCAAAGACAATCAGCAGAAA-3′
*TBXT*-R	5′-CACAAAAGGAGGGGCTTCACTA-3′
*MIXL1*-F	5′-TGCTTTCAAAACACTCGAGGAC-3′
*MIXL1*-R	5′-GAGTGATCGAAGTAACAGGTGC-3′
*SP5*-F	5′-GAGATTTGAAACAGTGCTCGGG-3′
*SP5*-R	5′-GGAGCTGAAGACAAAAGCAACA-3′
*SOX1*-F	5′-GGCCAAGGTAACACTCATCGTA-3′
*SOX1*-R	5′-ACCCTGTGATTTGGGAAGTGAA-3′
*PAX6*-F	5′-GCGGGTGACAAAATAGTTGTCTT-3′
*PAX6*-R	5′-GCCAGGATGTCAAATCTCTCCA-3′
*DLX1*-F	5′-ATGCACTGTTTACACTCGGC-3′
*DLX1*-R	5′-GACTGCACCGAACTGATGTAG-3′
*FOXG1*-F	5′-CGTTCAGCTACAACGCGCTCAT-3′
*FOXG1*-R	5′-CAGATTGTGGCGGATGGAGTTC-3′
*HES5*-F	5′-CCAAGGAGAAAAACCGACTG-3′
*HES5*-R	5′-AACTCCTGCTCCAGCAGCA-3′
*LHX2*-F	5′-ATGCTGTTCCACAGTCTGTCG-3′
*LHX2*-R	5′-GCATGGTCGTCTCGGTGTC-3′
*LHX9*-F	5′-GATGGAGCGCAGATCCAAGAC-3′
*LHX9*-R	5′-CCAGCAGATAGTACCTGTCCG-3′
*TBR1*-F	5′-TCTGAGCTTCGTCACAGTTTC-3′
*TBR1*-R	5′-GCTGTTGTAGGCTCCGTTG-3′
*ROBO1*-F	5′-TCCACACAGCAATAGCGAAG-3′
*ROBO1*-R	5′-CCTGTAACATGGGCTGGAGT-3′

### EdU assay

To assess cell proliferation rate, 5-ethynyl-20-deoxyuridine (EdU) cell proliferation kit (Sangon Biotech, E6072040100) was used. Cells cultured to around 70% confluency were incubated with medium containing EdU (10 μM) at 37 °C for 2 hours, fixed in 150 μL of 4% paraformaldehyde for 30 minutes at room temperature, incubated with 150 μL of 2 mg/mL glycine solution, and permeabilized with 300 μL of 0.5% Triton X-100 in PBS for 15 minutes at room temperature, after being washed with 1× PBS for 5 minutes. Cells were then stained with Hoechst 33342 for 10 minutes, mounted, and observed under Zeiss LSM 880 microscope to count EdU-positive cell rate. All images were processed by Image J.

### SSEA4 flow cytometry

The SSEA4 flow cytometry was used for measuring the stem cell maintenance ability. Cells were digested and stained with primary antibody, fluorescein isothiocyanate (FITC)-conjugated anti-SSEA4 antibody, followed by incubation on ice in the dark for 15 minutes. After treated with a FACS solution (containing 1% BSA in PBS), the mixture was centrifuged at 1200 rpm for 5 minutes at 4 °C, and the supernatant was removed, followed by resuspension of cell sediment in FACS buffer with 7AAD (Beyotime, ST515, diluted at a ratio of 1:1000) and incubation in darkness for 5 minutes on ice. Single-cell gating was performed using forward scatter area (FSC-A) against forward scatter height (FSC-H) to effectively separate out any aggregated cells. Dead cells were identified and removed from the single-cell group by excluding those that exhibited positive staining with Live/dead Blue. Negative threshold gates were defined with control samples (secondary antibodies alone).

### RNA-seq library construction, quality control, and sequencing

To construct RNA-seq libraries, total RNAs were extracted from about 1 million cells and were used as input material for RNA sample preparation using Fast RNA-seq Lib Prep Kit V2 (Cat.No.RK20306) following the manufacturer’s protocol. Briefly, poly-T oligo-attached magnetic beads were used to purify mRNA from total RNAs. Then, first strand cDNA was synthesized using random hexamer primer and M-MuLV Reverse Transcriptase (RNase H-), and second strand was performed using DNA Polymerase I and RNase H. The remaining overhangs were transformed into blunt ends by polymerase and exonuclease reactions. To become ready for hybridization, adaptors with hairpin loop structures were ligated after the 3′ ends of DNA fragments. The library fragments were purified using the AMPure XP technology to select cDNA fragments in 370-420 bp. Universal PCR primers, Index (X) Primer and Phusion High-Fidelity DNA polymerase were used for PCR, and the PCR products were purified.

Following the construction of the library, the library is diluted to 1.5 ng/μL, and first quantification is performed using Qubit following a detection of the inserted fragments. qRT-PCR was conducted to quantify the effective concentration in order to ensure the quality of the library after the inserted fragments were detected as expected.

Qualified libraries were pooled and sequenced on NovaSeq 6000 (Illumina) if they met the required effective library concentration and data amount.

### RNA-seq analysis

For the initial RNA-seq data, quality evaluation was performed using the FastQC tool (version 0.11.9). This step assessed the overall integrity of the RNA-seq fastq files and identified any potential issues. The bulk RNA-seq libraries were processed with paired-end sequencing. This was followed by trimming the sequences using the cutadapt software (version 4.1). The trimmed reads were aligned to the human genome (hg38) using the STAR aligner (version 2.7.10a) and organized with samtools (version 1.6), and the read counts were quantified against Gencode (version 41) annotations with featureCounts (version 2.0.1).

### Differential gene expression analysis

The differential gene expression was analyzed using DESeq2 (version 1.40.2) in the R programming environment (version 4.3.1). We normalized the gene count data from the RNA-seq analysis in DESeq2 and performed a variance stabilization transformation. To determine significant differential expression, we set a threshold of an adjusted *P*-value below 0.05 and an absolute log2FoldChange above 2. The findings were graphically represented using ggplot2 (version 3.4.4).

### Gene Set Enrichment Analysis

Firstly, the gene symbols from differentially expressed genes (DEGs) were translated to ENTREZ IDs using the org.Hs.eg.db package (version 3.17.0). The genes were ranked by their log2FoldChange values to compile a gene list. Gene Set Enrichment Analysis (GSEA) was then carried out using the gseGO function from the clusterProfiler package (version 4.9.0.2), encompassing all Gene Ontology (GO) categories, including biological processes, molecular functions, and cellular components. The analysis parameters included 1000 permutation tests, a gene set size range from 100 to 500, and a *P*-value cutoff of .75.

### iTRIBE analysis

The BAM files from the RNA-seq analysis were meticulously analyzed using the TRIBE algorithm to accurately identify A-to-I(G) edits. The sequence of YTHDF2-ADAR hESC is compared with the sequence of ADAR hESC. RNA-editing events are identified at loci where the edit threshold of at least 1% and a minimum read count of 20. TRIBE generated a comprehensive list of ADAR-mediated editing sites and identified genes that were potential targets of YTHDF2.

## Results

### Establishment of *YTHDF2* knock-out hESCs

**Figure 1.**
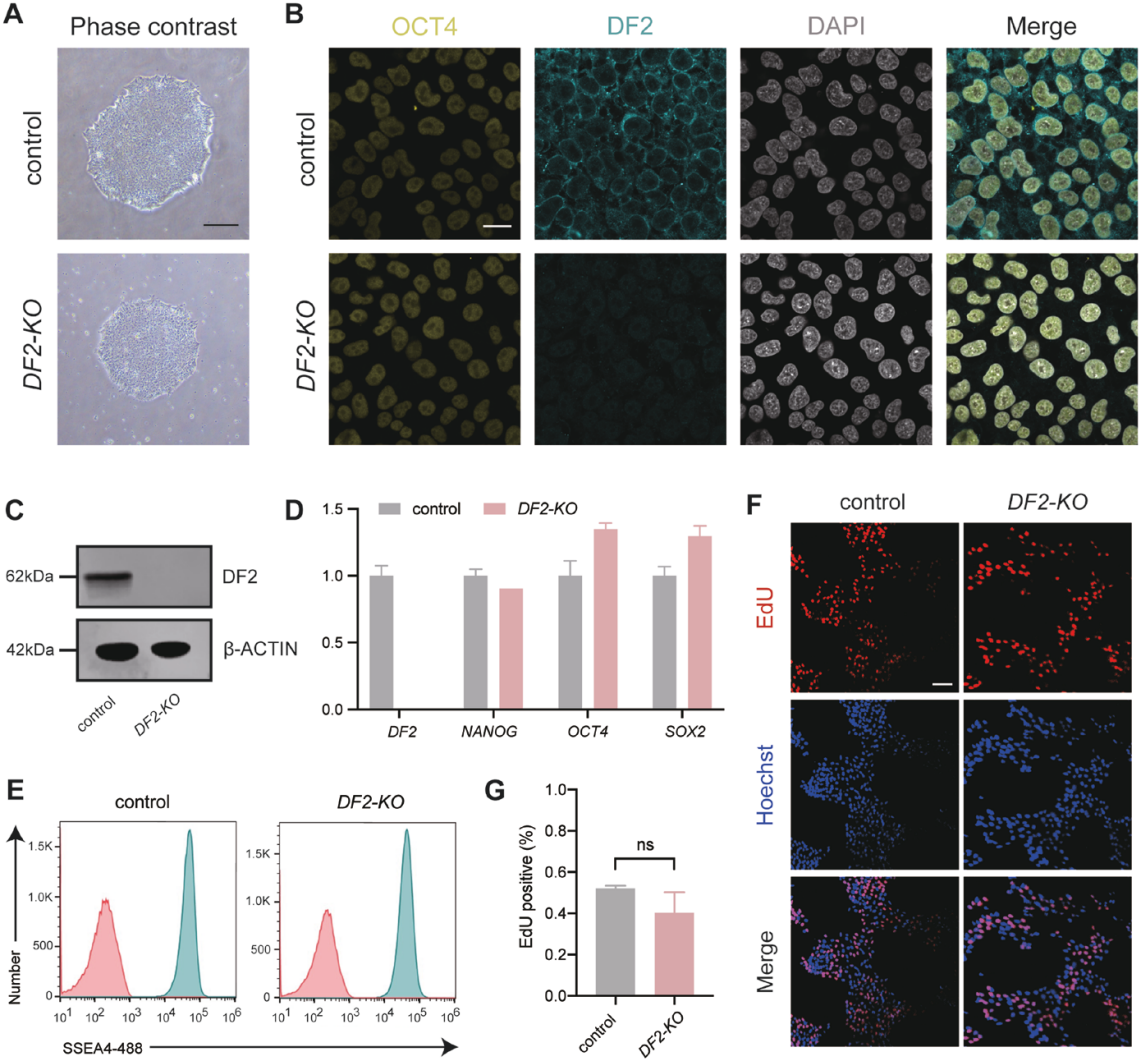
Generation of *YTHDF2* knock-out human embryonic stem cell (hESC) lines. (A) Phase-contrast images showing the morphology of control and *DF2-KO* hESCs. Scale bar: 200 μm. (B) Immunofluorescence showing the expression of DF2 and OCT4 in control and *DF2-KO* hESCs. DAPI is counterstained to indicate nuclei. Scale bar: 20 μm. (C) Western blot against DF2 in control and *DF2-KO* hESCs. β-ACTIN is used as a loading control. (D) RT-qPCR showing the expression level of *DF2*, *NANOG*, *OCT4*, and *SOX2* in control and *DF2-KO* hESCs. (E) Flow cytometry of SSEA4 on control and *DF2-KO* hESCs. (F, G) EdU incorporation assay of control and *DF2-KO* hESCs. Scale bar: 100 μm.

To explore the functions of the m^6^A reader *YTHDF2* (*DF2*) in the regulation of hESCs, we generated hESCs with knock-out of *DF2* (*DF2-KO*) using the CRISPR/Cas9 system.^19-21^ Two sgRNAs targeting the start and stop codons were designed to delete the whole genomic region of *DF2* (Supplementary Figure S1A). Given that the genomic region of *DF2* is large (~32 kb in length), we chose the gene replacement strategy that the cassettes with expression of antibiotic genes were designed to replace the genomic region of *DF2* (Supplementary Figure S1A). By selection with antibiotic drugs and genotyping PCR, hESCs with complete knock-out of *DF2* were generated (Supplementary Figure S1B). Importantly, the morphology of the *DF2-KO* hESCs was comparable to control hESCs even after being cultured for more than 30 passages (Figure 1A), suggesting that *DF2* may not be necessary for the maintenance of hESCs. The deletion of *DF2* in *DF2-KO* hESCs was further confirmed by immunofluorescence, western blot, and qPCR (Figure 1B-D). Notably, the expression of pluripotency genes, such as *OCT4*, *NANOG*, and *SOX2*, is comparable to control hESCs (Figure 1B and D), further suggesting that *DF2* is not important for the self-renewal of hESCs. We also determined the expression of pluripotency marker SSEA4 by flow cytometry and discovered that *DF2-KO* hESCs were positive for SSEA4 similar to control hESCs (Figure 1E), supporting the conclusion that *DF2* is not essential for the self-renewal of hESCs. Next, we sought to determine the proliferation rate of *DF2-KO* hESCs by EdU incorporation assay and discovered that the proliferation of *DF2-KO* hESCs was comparable to control hESCs (Figure 1F and G). Taken together, these results indicate that *DF2* is not essential for the self-renewal of hESCs.

### Transcriptomic characterization of the *DF2-KO* hESCs

**Figure 2.**
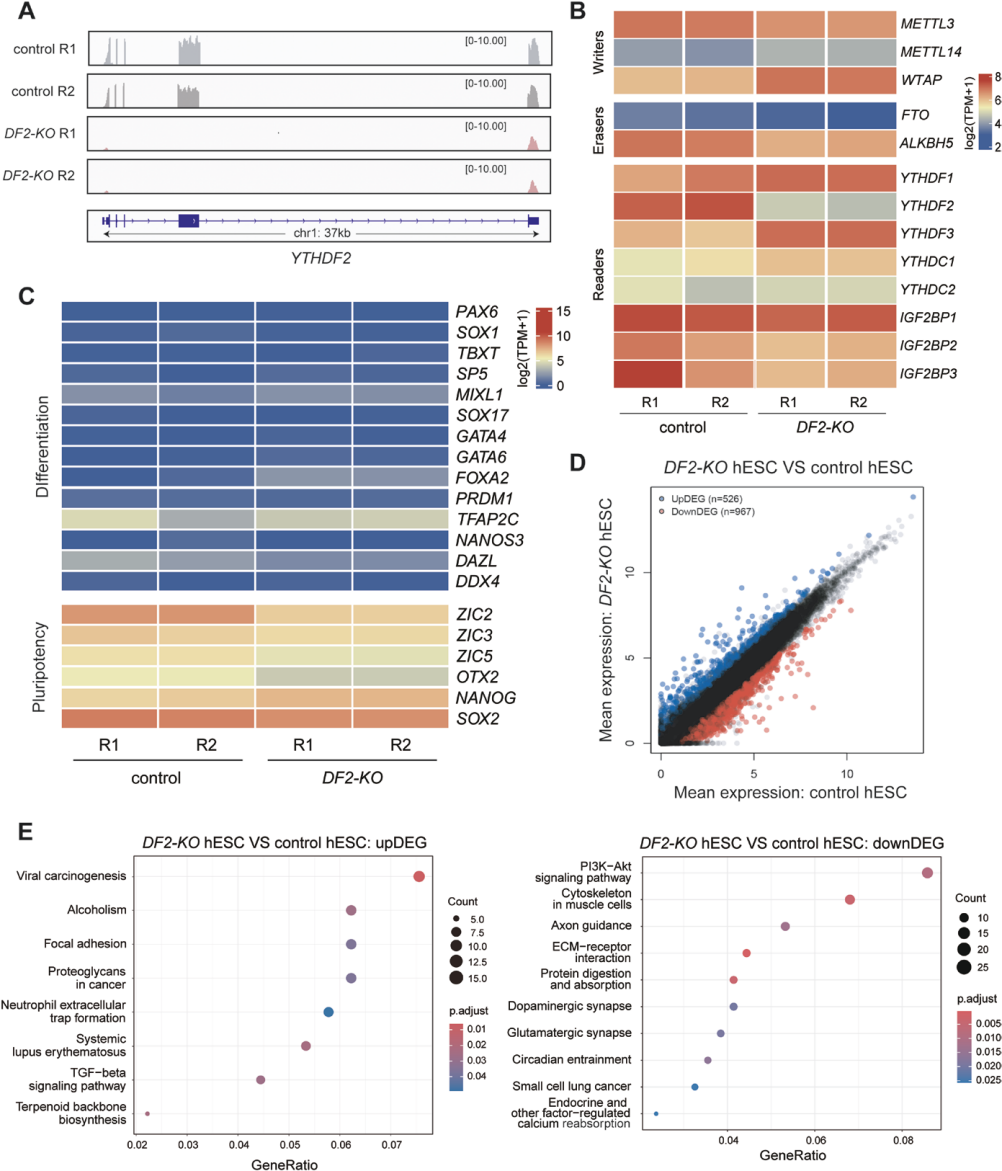
Transcriptomic analysis of *DF2-KO* human embryonic stem cells (hESCs). (A) Tracks showing the *DF2* mRNAs from RNA-seq samples of control and *DF2-KO* hESCs. (B) Heatmap showing the expression of m^6^A writers, erasers, and readers in control and *DF2-KO* hESCs. R: replicate. (C) Heatmap showing the expression of pluripotency- and differentiation-related markers in control and *DF2-KO* hESCs. (D) Scatterplot of differentially expressed genes (DEGs) in control and *DF2-KO* hESCs. (E) Kyoto Encyclopedia of Genes and Genomes (KEGG) analysis showing the biological terms enriched for genes upregulated (left) and downregulated in *DF2-KO* hESCs (right), compared to control hESCs.

Next, we performed RNA-seq of control and *DF2-KO* hESCs to explore the potential effects of *DF2-KO* on the transcriptome of hESCs. First, we confirmed that *DF2* was completely deleted (Figure 2A). We examined the expression of m^6^A writers, erasers, and readers in *DF2-KO* and control hESCs and discovered that the expression levels of most of these factors were not altered, however, the expression of *YTHDF3* (*DF3)* was upregulated in *DF2-KO* hESCs, suggesting that there might be compensation of *DF2* by *DF3* (Figure 2B). At the transcriptomic level, *DF2-KO* hESCs were similar to control hESCs (Supplementary Figure S2A and B and Supplementary Table 1), consistently with the above discovery that *DF2* is not essential for the self-renewal of hESCs. To further confirm this, we examined the expression of pluripotency and differentiation genes and discovered that the expression level of pluripotency genes was comparable in *DF2-KO* compared with control hESCs. Meanwhile, the differentiation-related genes were not ectopically activated (Figure 2C). There were 526 upregulated and 967 downregulated DEGs in *DF2-KO* compared with control hESCs (Figure 2D and Supplementary Table 2). Kyoto Encyclopedia of Genes and Genomes (KEGG) analysis revealed that the upregulated DEGs were enriched in the pathways of viral carcinogenesis, alcoholism, and focal adhesion, while the downregulated DEGs were enriched in PI3K-Akt signaling pathway and axon guidance (Figure 2E and Supplementary Table 3). GO analysis indicated that the up- and downregulated DEGs were enriched for terms of molecular functions such as protein heterodimerization activity, metal ion transmembrane transporter activity, and different channels activity (Supplementary Figure S2C and Supplementary Table 4). GSEA analysis revealed the enrichment of alcoholism, neutrophil extracellular trap formation, and endocrine carcinogenesis erythematosus calcium in *DF2-KO* cells at the hESC stage (Supplementary Figure S2D and E and Supplementary Table 5). Altogether, these observations confirm the conclusion that *DF2* is not essential for the self-renewal of hESCs at the transcriptomic level.

### Characterization of the functions of *DF2* in the differentiation of hESCs

**Figure 3.**
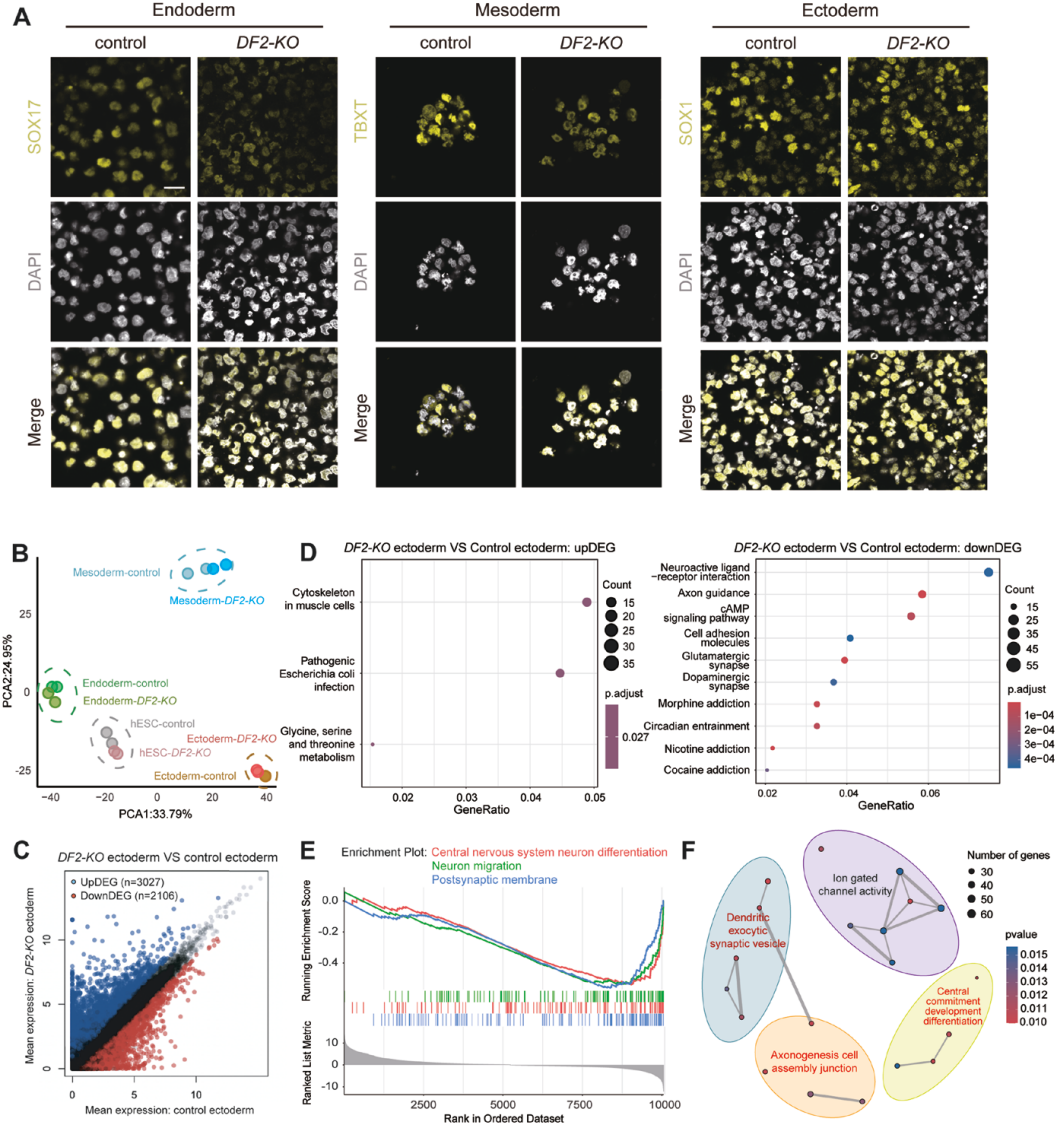
Functional analysis of DF2 in human embryonic stem cell (hESC) differentiation. (A) Immunofluorescence showing the expression of SOX17, TBXT, and SOX1 in control and *DF2-KO* hESC-derived endoderm, mesoderm, and ectoderm, respectively. (B) Principal component analysis (PCA) plot of control and *DF2-KO* hESCs, and hESC-derived endoderm, mesoderm, and ectoderm. (C) Scatterplot of differentially expressed genes (DEGs) in control and *DF2-KO* hESC-derived ectoderm. (D) Kyoto Encyclopedia of Genes and Genomes (KEGG) analysis showing the biological terms enriched for genes upregulated (left) and downregulated (right) in *DF2-KO* hESC-derived ectoderm, compared to control hESC-derived ectoderm. (E, F) Enrichment plot (left) and Emap plot (right) of Gene Set Enrichment Analysis (GSEA) analysis on KEGG terms showing the biological terms enriched for DEGs in *DF2-KO* hESC-derived ectoderm, compared to control hESC-derived ectoderm.

Next, we sought to determine whether *DF2* is involved in the regulation of hESC differentiation. To achieve this, we differentiated hESCs towards endoderm, mesoderm, and ectoderm.^22^ We confirmed the differentiation by immunofluorescence with germ layer-specific markers such as SOX17 for endoderm, TBXT for mesoderm, and SOX1 for ectoderm (Figure 3A). We then performed qPCR to detect the expression levels of the markers for pluripotency, endoderm, mesoderm, and ectoderm. These marker genes of the 3 germ layers were expressed at comparable levels between control and knock-out cells (Supplementary Figure S3A). To explore the potential functions of *DF2* in the differentiation of hESCs at the transcriptomic level, we performed RNA-seq of control and *DF2-KO* hESC-derived endodermal, mesodermal, and ectodermal cells (Supplementary Table 1). The *DF2-KO* and control cells were clustered together in the corresponding germ layers in the principal component analysis (PCA) plot (Figure 3B), consistent with the similar expression levels of the corresponding germ layer markers in *DF2-KO* cells compared with control cells (Supplementary Figure S3B). This is further confirmed by correlation and transcriptome-wide heatmaps (Supplementary Figure S3C-E and Supplementary Table 6). For hESC-derived endodermal cells, we identified 800 upregulated genes and 703 downregulated genes (Supplementary Table 7). KEGG analysis indicated that these genes were mainly enriched in terms such as neurodegeneration-multiple diseases, and MAPK signaling pathway (Supplementary Figure S4A and Supplementary Table 8). GO term analysis suggested that the up- and downregulated DEGs were enriched for terms of the growth factor activity, DNA-binding transcription activator activity, RNA polymerase II-specific, and extracellular matrix structural constituent (Supplementary Figure S4B and Supplementary Table 9). Furthermore, GSEA analysis revealed the functional enrichment of *DF2-KO* endodermal cells in animal organ morphogenesis, extracellular matrix, and negative regulation of multicellular organismal process (Supplementary Figure S4C and D and Supplementary Table 10). These observations suggest that *DF2* may play limited roles in endoderm differentiation. Interestingly, we observed a higher number of DEGs in *DF2-KO* mesodermal and ectodermal cells compared to control cells. In mesoderm, there were 1699 upregulated genes and 1972 downregulated genes, mainly enriched in ribosome biogenesis in eukaryotes and PI3K-Akt signaling pathway, respectively (Supplementary Figure S5A and Supplementary Tables 11 and 12). GO analysis indicated that the up- and downregulated DEGs were enriched for terms of catalytic activity acting on RNA and actin binding, respectively (Supplementary Figure S5B and Supplementary Table 13). GSEA analysis revealed the functional enrichment of *DF2-KO* ectodermal cells in immune response functions, such as adaptive immune response and response to bacterium (Supplementary Figure S5C and D and Supplementary Table 14). Notably, 3027 up- and 2106 downregulated DEGs were identified in *DF2-KO* ectodermal cells compared to control cells (Figure 3C and Supplementary Table 15). The upregulated genes were enriched for terms such as GTPase regulator activity, nucleoside-triphosphatase regulator activity, channel activity, and passive transmembrane transporter activity in GO term analysis (Supplementary Figure S6A and Supplementary Table 16). The downregulated DEGs were enriched in the pathway of neuroactive ligand-receptor interaction and axon guidance, implying that *DF2* may play a vital role in ectodermal stage by affecting neural-related pathways (Figure 3D and Supplementary Table 17). Consistently, GSEA analysis revealed the functional enrichment of *DF2-KO* ectodermal cells in neural development, such as central nervous system neuron differentiation and neuron migration (Figure 3E and F and Supplementary Table 18). Taken together, these data demonstrate the potential regulatory functions of *DF2* in neural development.

### Characterization of the functions of *DF2* in neural differentiation

Next, we focused on the functions of *DF2* in neural differentiation. We first defined a subset of genes that were upregulated during hESC to neuroectoderm differentiation by comparing the control ectodermal cells with the control hESCs. Then, we compared the *DF2-KO* ectodermal cells to the *DF2-KO* hESCs, resulting in a subset of genes upregulated during hESC to neuroectoderm differentiation in *DF2-KO* conditions. By analyzing the intersection of these 2 subsets of upregulated DEGs, we identified 1223 genes that were failed to be upregulated in *DF2-KO* ectodermal cells that might represent the functional genes for *DF2* in regulating neural development such as DLX1, FOXG1, HES5, LHX2, LHX9, and TBR1 (Supplementary Figure S6B and Supplementary Table 19). Dlx1 is involved in differentiation of a subset of GABAergic neurons of the basal ganglia and cerebral cortex.^23^ Foxg1 is an important factor in telencephalon development.^24^ Hes5 inhibits the differentiation of neural stem cells as a downstream effector of Notch signaling.^25^ Lhx2 is highly expressed in the ventricular zone of the dorsal telencephalon and prevented premature neuronal differentiation.^26^ Lhx9 is reported to function in neurogenesis with Lhx2.^27^ Tbr1 contributes to the development of preplate and layer VI neurons, axon guiding, and the proper identity of projection neurons.^28^ To validate the functions of *DF2* in hESC to neuroectoderm development, we overexpressed *DF2* in *DF2-KO* cells and discovered the re-expression of the above genes (Supplementary Figure S6C). These results further support the conclusion that *DF2* is involved in the regulation of hESC to neuroectoderm differentiation.

**Figure 4.**
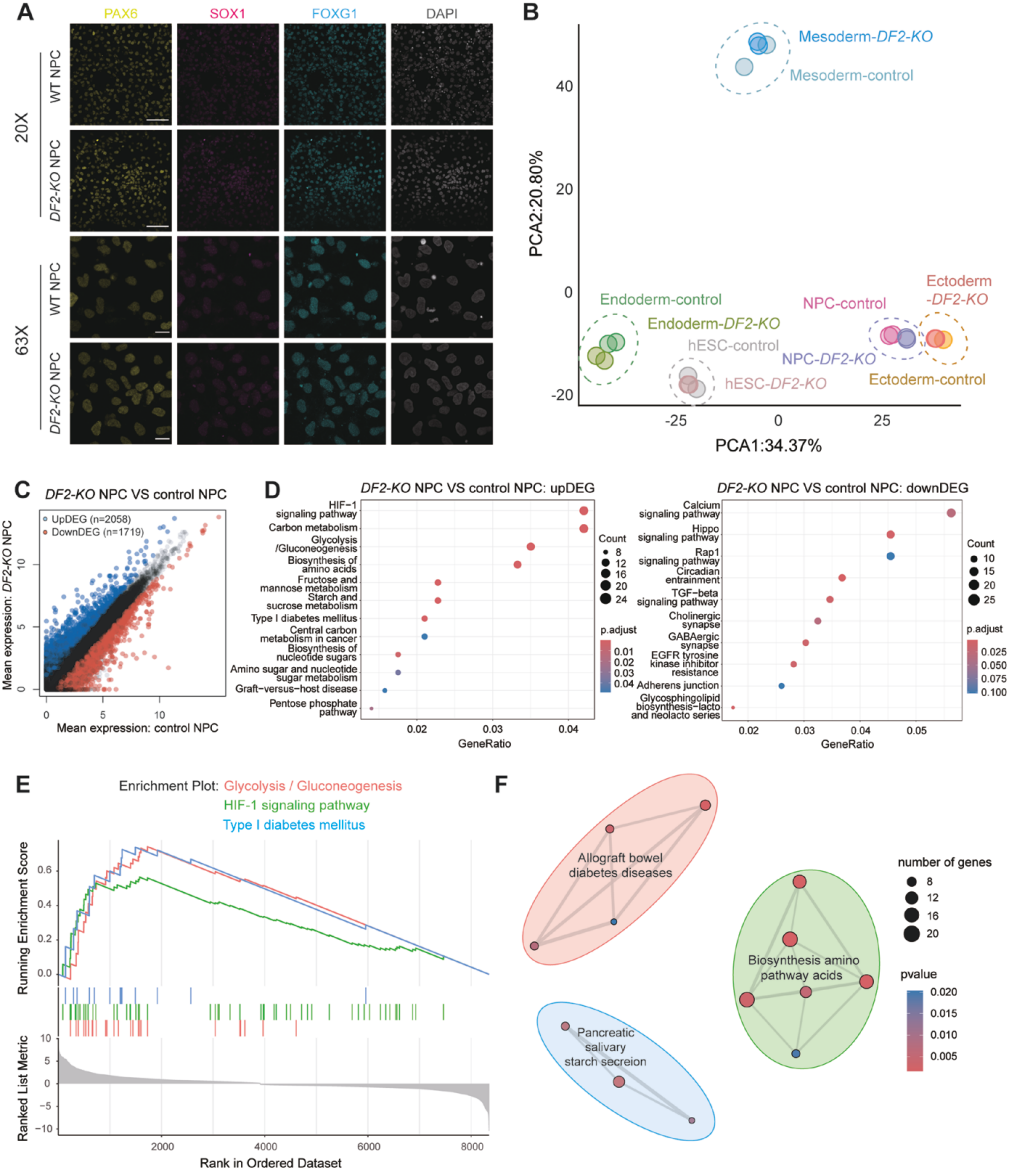
Functional analysis of DF2 in neural progenitor cells (NPCs). (A) Immunofluorescence showing the expression of PAX6, SOX1, and FOXG1 in control and *DF2-KO* human embryonic stem cell (hESC)-derived NPCs. (B) Principal component analysis (PCA) plot of control and *DF2-KO* hESCs, and hESC-derived endoderm, mesoderm, ectoderm, and NPCs. (C) Scatterplot of differentially expressed genes (DEGs) in control and *DF2-KO* hESC-derived NPCs. (D) Kyoto Encyclopedia of Genes and Genomes (KEGG) analysis showing the biological terms enriched for genes upregulated (left) and downregulated (right) in *DF2-KO* hESC-derived NPCs, compared to control hESC-derived NPCs. (E, F) Enrichment plot (left) and Emap plot (right) of Gene Set Enrichment Analysis (GSEA) analysis on KEGG terms showing the biological terms enriched for DEGs in *DF2-KO* hESC-derived NPCs, compared to control hESC-derived NPCs.

To further explore the functions of *DF2* in neural development, we employed an induction system for differentiation towards NPCs from hESCs. We first examined the expression of NPC markers including SOX1, PAX6, and FOXG1 (Figure 4A). To further explore the potential functions of YTHDF2 in hESC to NPC differentiation, we performed RNA-seq (Supplementary Table 1). Interestingly, *DF2-KO* and control NPCs were clustered together in the PCA plot (Figure 4B). Moreover, *DF2-KO* and control NPCs exhibited high similarity by correlation and transcriptome-wide heatmaps (Supplementary Figure S7A and B and Supplementary Table 6). Next, we examined the expression of pluripotency and NPC markers and discovered the decreased expression of pluripotency genes and upregulation of NPC genes in both *DF2-KO* and control cells (Supplementary Figure S7C). Finally, we examined the DEGs between *DF2-KO* and control NPCs. There were 2058 upregulated and 1719 downregulated DEGs (Figure 4C and Supplementary Table 20). GO term analysis indicated that the upregulated DEGs were enriched for terms such as lyase activity, organic anion transmembrane transporter activity, GTPase regulator activity, and nucleoside-triphosphatase regulator activity (Supplementary Figure S7D and Supplementary Table 21). Importantly, the downregulated DEGs were enriched for GABAergic synapse and EGFR tyrosine kinase inhibitor resistance, which are closely related to neural functions (Figure 4D and Supplementary Table 22), demonstrating the involvement of *DF2* in neural development. GSEA analysis revealed the functional enrichment of terms such as glycolysis and HIF-1 signaling pathway in *DF2-KO* NPCs (Figure 4E and F and Supplementary Table 23). Taken together, these results indicate that DF2 is involved in the regulation of neural development.

### Identification and analysis of DF2 downstream targets

**Figure 5.**
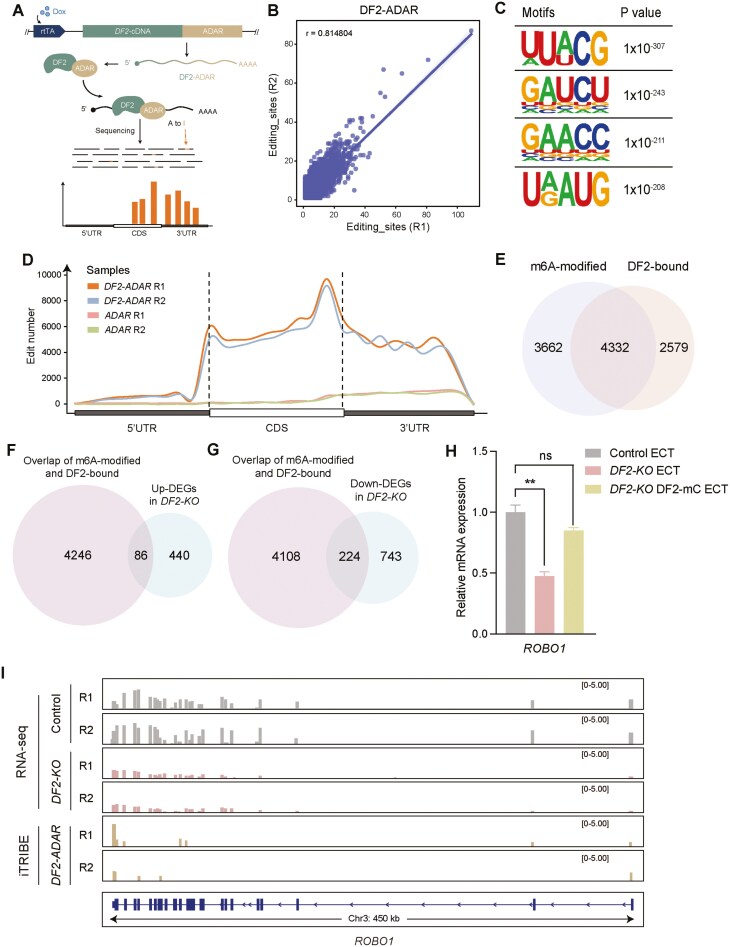
DF2 regulates human embryonic stem cell (hESC) to ectoderm differentiation through *ROBO1*. (A) Schematic illustration of the strategy for iTRIBE. (B) Correlation analysis on the number of edits on editing sites between the 2 biological replicates of RNA-seq on *DF2-ADAR* hESCs. (C) Motif analysis of DF2-binding site. (D) The distribution of DF2-binding sites across gene regions. CDS = coding sequence; UTR = untranslated regions. (E) Venn diagram showing the overlap of m^6^A-modified genes^9^ and DF2-bound genes. (F, G) Venn diagrams showing the overlap of the 4332 overlapping genes from (E) to upregulated (F) and downregulated (G) differentially expressed genes DEGs of *DF2-KO* compared with control. (H) RT-qPCR on ROBO1 for control, *DF2-KO*, and *DF2-rescue* hESC-derived ectoderm. **P < .01; ns, *P* > .05. (I) Tracks showing the expression of *ROBO1* in control and *DF2-KO* hESCs by RNA-seq and binding of DF2-ADAR to *ROBO1* by iTRIBE.

Next, we sought to understand the mechanisms by which DF2 regulates neural development by capturing the key target mRNAs bound by DF2, given that DF2 is an m^6^A reader protein. To achieve this, we conducted the iTRIBE of DF2.^29,30^ DF2 was fused with the catalytic domain of the RNA-editing enzyme Adenosine Deaminase Acting on RNA (ADAR) from Drosophila (DF2-ADAR) and was expressed in hESCs in a Doxycycline (Dox)-inducible manner. With the pulse overexpression of DF2-ADAR in hESCs with the administration of Dox, the DF2-bound mRNAs could be captured by identification of A to G conversion (due to the A to I editing by ADAR) (Figure 5A).^29,30^ We first confirmed the expression of DF2-ADAR by western blot (Supplementary Figure S8A). Next, we performed RNA-seq of *DF2-ADAR* and control hESCs. The expression of ADAR was detected in *DF2-ADAR* hESCs but not in control hESCs (Supplementary Figure S8B). We then compared the transcriptomes of *DF2-ADAR* and control hESCs and found that they were comparable to each other by correlation analysis (Supplementary Figure S8C) and clustered together in PCA plot (Supplementary Figure S8D). These observations suggest that the pulse overexpression of DF2-ADAR has no dramatic effects on the transcriptomes of hESCs, indicating these samples could be applied to analyze the target mRNAs of DF2.

We determined the mRNA targets of DF2 by examination of A to G conversions and found that the 2 biological replicates were highly correlated with each other (Figure 5B), forming the basis for further analysis. We then analyzed the possible motifs in mRNAs that might be recognized by DF2 in hESCs based on iTRIBE (Figure 5C). The binding regions of mRNAs by DF2 were enriched in the CDS and 3′UTR, peaking at the sites around stop codons (Figure 5D), similar to the m^6^A modification patterns in mRNAs.^31,32^ Given that DF2 is a well-characterized m^6^A reader protein, we next overlapped the DF2-bound and m^6^A-modified mRNAs^9^ for identifying the potential key downstream effectors of DF2. There were 4332 genes that were both bound by DF2 and modified by m^6^A in hESCs (Figure 5E and Supplementary Table 24). We then overlapped these DF2-bound and m^6^A-modified genes to the upregulated and downregulated DEGs in *DF2-KO* compared with control hESCs. However, there were only 86 upregulated DEGs and 224 downregulated DEGs bound by DF2 and modified by m^6^A at mRNA level (Figure 5F and G and Supplementary Table 25).

These 86 upregulated DEGs were enriched for terms such as transforming growth factor beta receptor binding and SMAD binding, while the 224 downregulated DEGs were enriched for terpenoid backbone biosynthesis and cytoskeleton in muscle cells in KEGG (Supplementary Figure S8E and Supplementary Table 26) and enriched for transforming growth factor beta receptor binding, ribosome binding, and transcription coactivator activity, and exopeptidase activity in GO term analysis (Supplementary Figure S8F and Supplementary Table 27). Interestingly, a subset of these 244 downregulated genes were related to neurogenesis, for example, *FGFR1*, *OTX2*, *EGR1*, *ROBO1*, *MSI1*, *CDKN1A*, *CDKN1B*, and *ZIC2* (Supplementary Table 25). *ROBO1* is a transmembrane receptor protein which has been shown to be involved in axonal repulsion,^33,34^ neuronal migration,^35^ and axon guidance.^36^ More importantly, *ROBO1* mRNAs were bound by DF2 determined by iTRIBE (Figure 5H).

To confirm the m^6^A-dependent regulation of DF2 on *ROBO1* mRNAs, we constructed the hESC line with overexpression of dCas13b-ALKBH5 and crRNA-ROBO1 (*ALKBH5-ROBO1*) (Supplementary Figure S9A). With the crRNA targeting the m^6^A on *ROBO1* mRNAs,^9^ dCas13b-ALKBH5 specifically binds to *ROBO1* mRNA for site-specific removal of m^6^A modification. If the m^6^A modification in *ROBO1* mRNAs is critical for the DF2-mediated regulation, we would expect that site-specific removal of m^6^A modification on *ROBO1* mRNAs would phenocopy *DF2-KO* condition. Importantly, we found that site-specific removal of m^6^A modification resulted in the downregulation of *ROBO1* mRNAs (Supplementary Figure S9B). To further explore the potential functions of *ROBO1* in neuroectodermal differentiation, we performed RNA-seq of ectodermal cells derived from *ALKBH5-ROBO1* and *ALKBH5-*control (overexpression of dCas13b-ALKBH5 without crRNA) hESCs (Supplementary Table 1). *ALKBH5-ROBO1* and *ALKBH5-*control ectodermal cells were clustered together with other ectoderms in the PCA plot (Supplementary Figure S9C) and show highly similarity by correlation and transcriptome-wide heatmaps (Supplementary Figure S9D and E and Supplementary Table 6). There were 9 upregulated and 20 downregulated DEGs comparing *ALKBH5-ROBO1* and *ALKBH5-*control ectodermal cells (Supplementary Figure S9F and Supplementary Table 28). About 33.3% (3 out of 9) of the upregulated DEGs were overlapped with the upregulated DEG of *DF2-KO* ectoderm, but importantly 65.0% (13 out of 20) of downregulated DEGs were overlapped with the downregulated DEGs of *DF2-KO* ectoderm (Supplementary Figure S9G and Supplementary Table 29). Notably, most of the overlapping downregulated DEGs (10 out of 13), such as NEUROG1 and EOMES, were related to neural development (Supplementary Table 29). Furthermore, GSEA analysis indicates the enrichments of these DEGs for brain development, central nervous system development, and nervous system development (Supplementary Figure S9H). Taken together, these results suggest that DF2 may act through *ROBO1* to regulate the differentiation of hESCs towards ectodermal lineage.

## Discussion

In this study, we focused on the m^6^A reader DF2 in regulating hESCs. By transcriptomic analysis of *DF2-KO* hESCs and hESC-derived endodermal, mesodermal, neuroectodermal cells, and NPCs, we discovered that *DF2-KO* damages the potential of hESC differentiation towards neuroectodermal lineage, probably via m^6^A-dependent regulation of *ROBO1*. Our iTRIBE analysis indicates that DF2 binds to *ROBO1* mRNAs, which are m^6^A-modified and downregulated in *DF2-KO* hESCs. This study establishes the m^6^A-mediated epitranscriptomic regulation in neuroectodermal differentiation, emphasizing the critical roles of m^6^A readers in developmental orchestration.

The YTHDF protein family members share a high degree of sequence similarity, and one study has reported the context-dependent functional compensation among *Ythdf1*, *Ythdf2*, and *Ythdf3* in mouse ESCs.^11^ Another study demonstrates that m^6^A sites are bound by all YTHDFs with redundant functions in degrading mRNAs.^37,38^ In this study, the upregulation of *YTHDF1* (*DF1*) and *DF3* was observed in *DF2-KO* hESCs (Figure 2B). Whether *DF1* and *DF3* are redundant to *DF2* for regulating neural development warrants further investigation. We also observed that DF2 binds to about 54.1% of m^6^A-modified mRNAs (Figure 5E), but not all mRNAs with m^6^A modification. This could be due to different roles of YTHDF family members in regulating hESC differentiation or due to the iTRIBE technique that might not capture all the mRNAs bound by DF2. Further research is needed to identify the target mRNAs bound by YTHDF family members, forming the basis for uncovering the functions of m^6^A modification and YTHDF family of m^6^A readers in regulating neural development.

*DF2* has been shown to be involved in the regulation of mouse neurogenesis,^12^ suggesting that the functions of the m^6^A reader *DF2* in neural development are conserved. *DF2-KO* mice died at the late embryonic stage, exhibiting defects in neural stem/progenitor cell (NPSC) self-renewal and spatiotemporal generation of neurons.^12^ Moreover, *DF2* is involved in various neuro-related diseases. *DF2* has been found to be significantly upregulated in ischemic stroke (IS) patients and showed significant association with IS by univariate logistic regression analysis.^39^*DF2* might accelerate this process by regulating matrilin-3.^40^ Additionally, *DF2* may be involved in the abnormal degradation of SIRT6 and Nrf2 caused by a lower level of FTO in IS models.^41,42^*DF2* has also been reported to be involved in Parkinson’s disease, dementia, amyotrophic lateral sclerosis, and inflammatory pain.^43-47^ However, the functions of *DF2* in human neuroectodermal development remain largely unknown. A recent study using siRNA-mediated knockdown of *DF2* (*DF2-KD*) in human induced pluripotent stem cells (hiPSCs) discovered that DF2 destabilized neural-specific mRNAs in hiPSCs to prevent neural differentiation.^48^ While our study revealed that DF2 is not required for the maintenance of hESCs, but for neuroectodermal differentiation. Some of the upregulated genes in *DF2-KD* hiPSCs^48^ (*NGFR*, *TSPYL4*, *TOR1A/P2*, *LINGO1*) were upregulated in *DF2-KO* hESCs. Some of the upregulated genes in *DF2-KD* hiPSCs^48^ (*ST6GALNAC4*, *FBLN2*, *CHAC1*, *WDR62*, *NLGN3*, *SYNGAP1*, *OPCML*, *EPHA8*, *AKT1*) were downregulated in *DF2-KO* hESCs (Supplementary Figure S9I). This may be due to the different techniques employed (siRNA-mediated knockdown compared with CRISPR/Cas9-mediated knockout) and/or different cell lines and culture/differentiation conditions. Nonetheless, *ROBO1* was downregulated in both *DF2-KO* hESCs and *DF2-KD* hiPSCs (Supplementary Figure S9J and K), indicating that *ROBO1* may represent a critical downstream effector for mediating m^6^A-dependent regulation of neural development.

Notably, *ROBO1* is involved in the balance of proliferation and differentiation of NPSCs, and *ROBO1* deficiency leads to an increased number of basally located progenitors in the neocortex in mice.^49^ In addition, *ROBO1* regulates the migration of neural cells. In *ROBO1-KO* mice, the interneurons are found in the striatum due to the defects of neural migration.^50,51^*ROBO1* has also been associated with neurodevelopmental disorders such as dyslexia^52,53^ and autism.^54^ In this study, we discovered that removing m^6^A modifications in *ROBO1* mRNAs by dCas13 system leads to the downregulation of neural differentiation-related genes (Supplementary Figure S9). Therefore, the m^6^A-DF2-*ROBO1* axis we characterized in this study may be critical for understanding the developmental regulation of neurogenesis and the pathogenesis of the related diseases.

As the most abundant internal modification in mRNAs, m^6^A broadly affects the central nervous system, regulating self-renewal of NPSCs, proliferation of glioma cells, development of brain, and growth of synapses.^55^ Knockout of the m^6^A writers *METTL3* or *METTL14* leads to impaired NPSC differentiation and prolonged cell cycle progression of radial glia in both mice and humans.^56^ Furthermore, m^6^A-mediated regulation was shown to be involved in learning and memory in mice. The m^6^A reader *YTHDF1* promotes the translation of the target mRNAs in response to neuronal stimuli in the adult mice, which in turn facilitates learning and memory.^57^ To be noted, DF2 is widely discovered to be correlated with the degradation of m^6^A modified mRNAs.^58^ However, our results suggest that DF2 regulates the stabilization of *ROBO1* mRNAs. Although our dCas13-based m^6^A removal results support the direct regulation of m^6^A modification for stabilizing *ROBO1* mRNAs, we could not fully exclude the possibility that DF2 may indirectly regulate *ROBO1* mRNAs. Further analysis of the diverse targets of YTHDF2 will help to further clarify the roles of YTHDF2 in stem cell differentiation. Moreover, m^6^A readers may play m^6^A-independent roles for RNA regulation. For example, YTHDC2 regulates the meiotic process via an m^6^A-independent manner, based on the discovery of low overlap between YTHDC2-bound mRNAs and m^6^A-modified mRNAs, and point mutations disrupting m^6^A-binding in YTHDC2 have no effect on meiosis in mice.^59^ Whether DF2 also regulates *ROBO1* mRNAs in an m^6^A-independent manner warrants further investigation. Overall, m^6^A-mediated post-transcriptional modulation represents an essential level of regulation of neurogenesis in the brain from embryonic to adult stages. Our discovery of the m^6^A reader *DF2* in the m^6^A-mediated regulation emphasizes the importance of m^6^A modification in neurogenesis. Future work using the hESC-derived neural organoids will facilitate the further interrogation of the functions of DF2 in human neurogenesis and provide new insights into the related neural diseases.

## Supplementary Material

sxaf032_suppl_Supplementary_Figure_S1

sxaf032_suppl_Supplementary_Figure_S2

sxaf032_suppl_Supplementary_Figure_S3

sxaf032_suppl_Supplementary_Figure_S4

sxaf032_suppl_Supplementary_Figure_S5

sxaf032_suppl_Supplementary_Figure_S6

sxaf032_suppl_Supplementary_Figure_S7

sxaf032_suppl_Supplementary_Figure_S8

sxaf032_suppl_Supplementary_Figure_S9

sxaf032_suppl_Supplementary_Tables_S1-S29

## Data Availability

All sequencing data supporting the findings of this study have been deposited in the NCBI Gene Expression Omnibus (GEO) under accession number GSE268806. The data for control hESC, and hESC-derived endoderm, mesoderm, and ectoderm cells are available under accession number GSE266418. The data for control ADAR samples can be found under accession number GSE244757. The m^6^A-modified gene list was obtained from accession number GSE52600.

## Code availability

The bioinformatics analysis codes have been deposited at Zenodo (https://doi.org/10.5281/zenodo.11565833) and are publicly accessible as of the publication date. Any additional information needed to reanalyze the data presented in this paper is available from the lead contact upon request.
