# Supplementary figures and images for: Transcriptomic analysis of the m6A reader YTHDF2 in the maintenance and differentiation of human embryonic stem cells
Source: Stem Cells. 2025 May 26;43(7):sxaf032. doi: 10.1093/stmcls/sxaf032 (PMC12202760; doi:10.1093/stmcls/sxaf032)

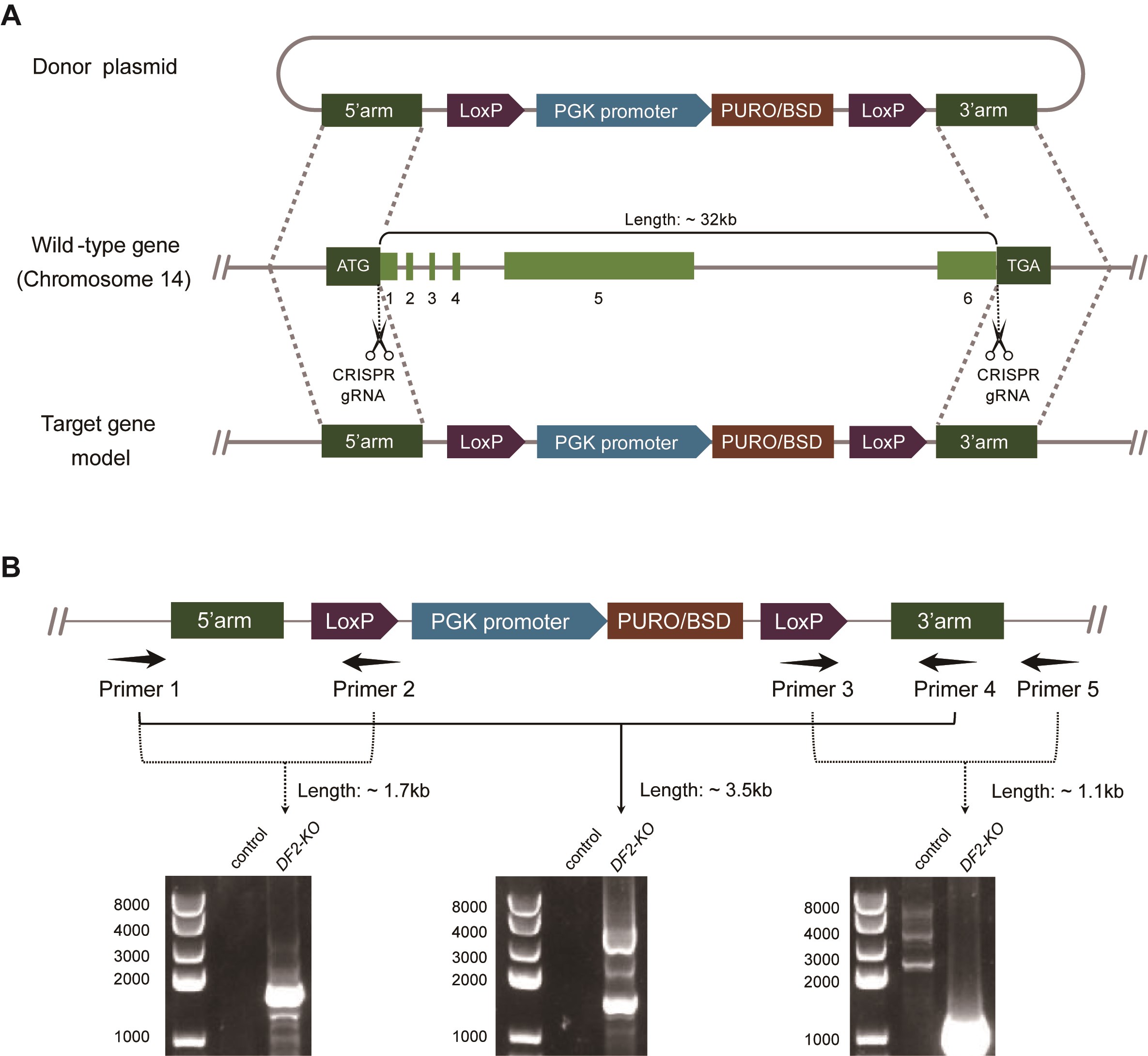

Supplement: sxaf032_suppl_Supplementary_Figure_S1 [file sxaf032_suppl_supplementary_figure_s1.jpeg]

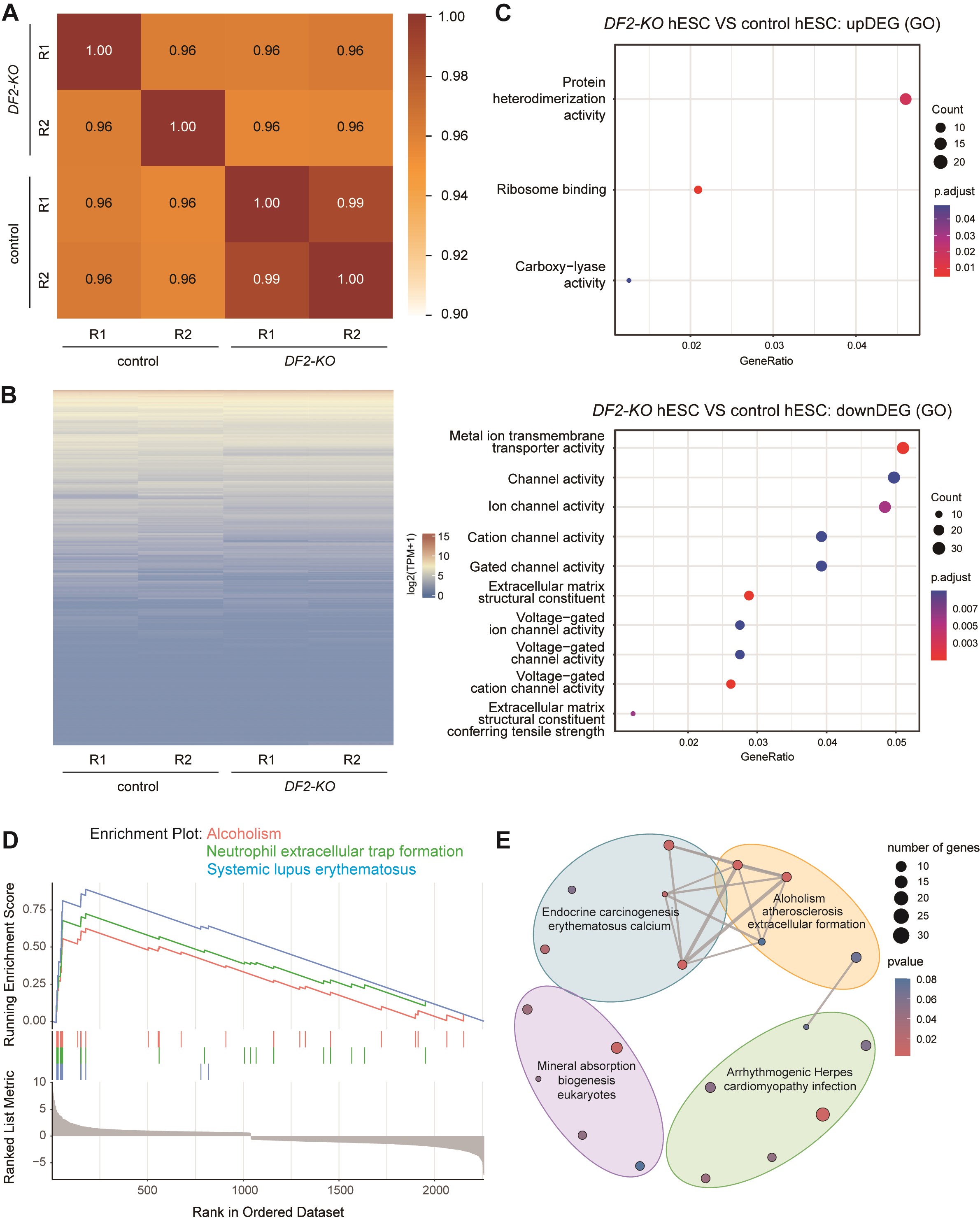

Supplement: sxaf032_suppl_Supplementary_Figure_S2 [file sxaf032_suppl_supplementary_figure_s2.jpeg]

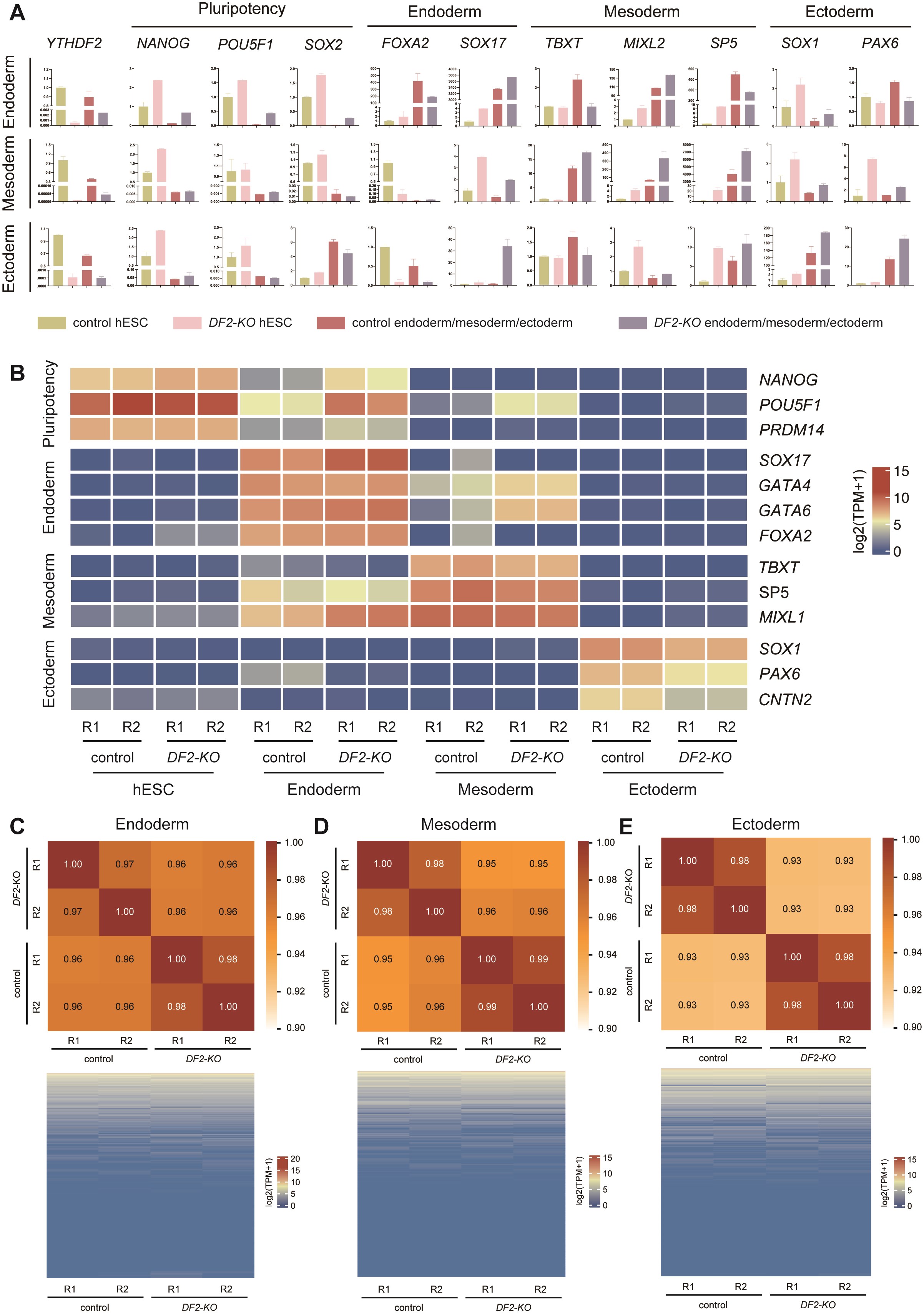

Supplement: sxaf032_suppl_Supplementary_Figure_S3 [file sxaf032_suppl_supplementary_figure_s3.jpeg]

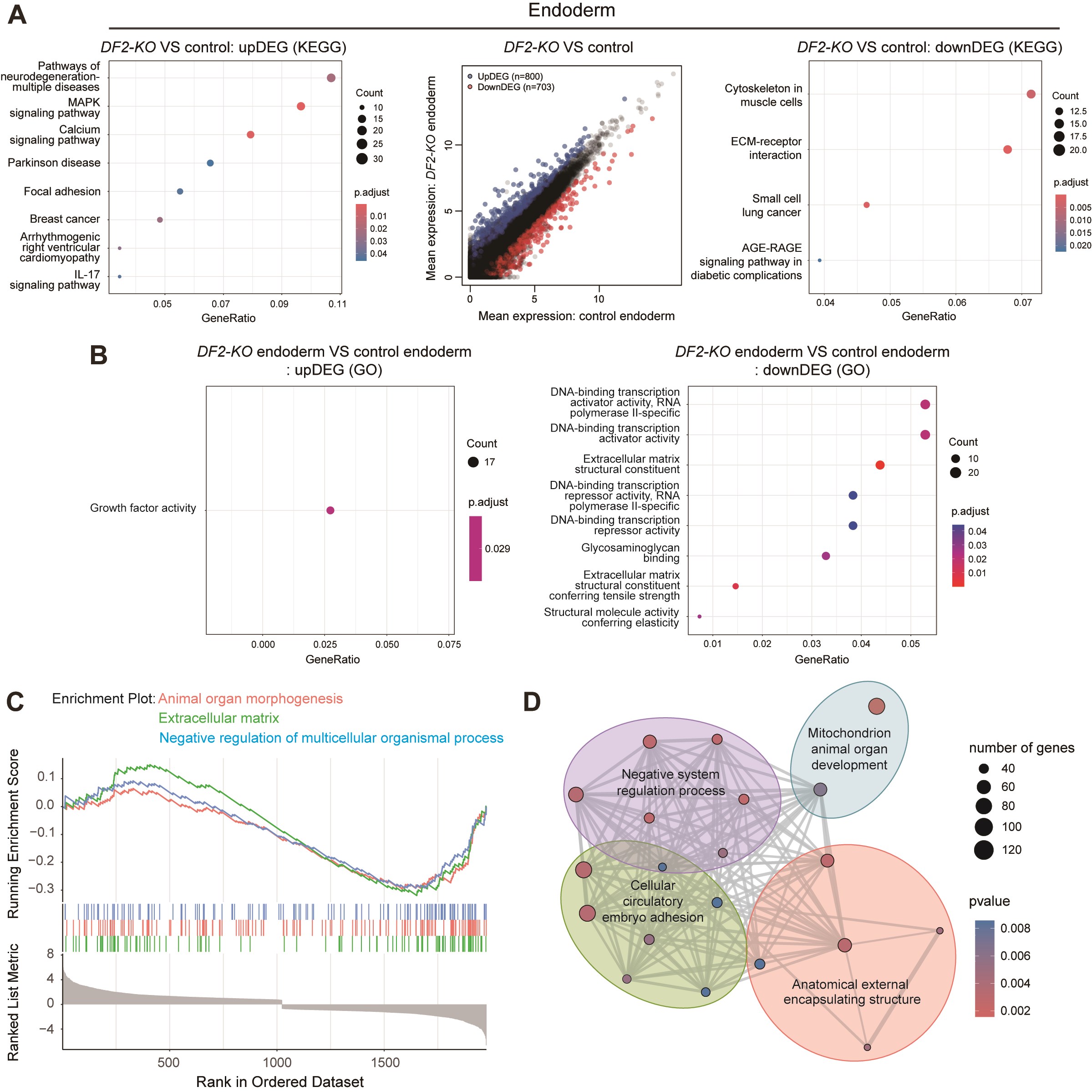

Supplement: sxaf032_suppl_Supplementary_Figure_S4 [file sxaf032_suppl_supplementary_figure_s4.jpeg]

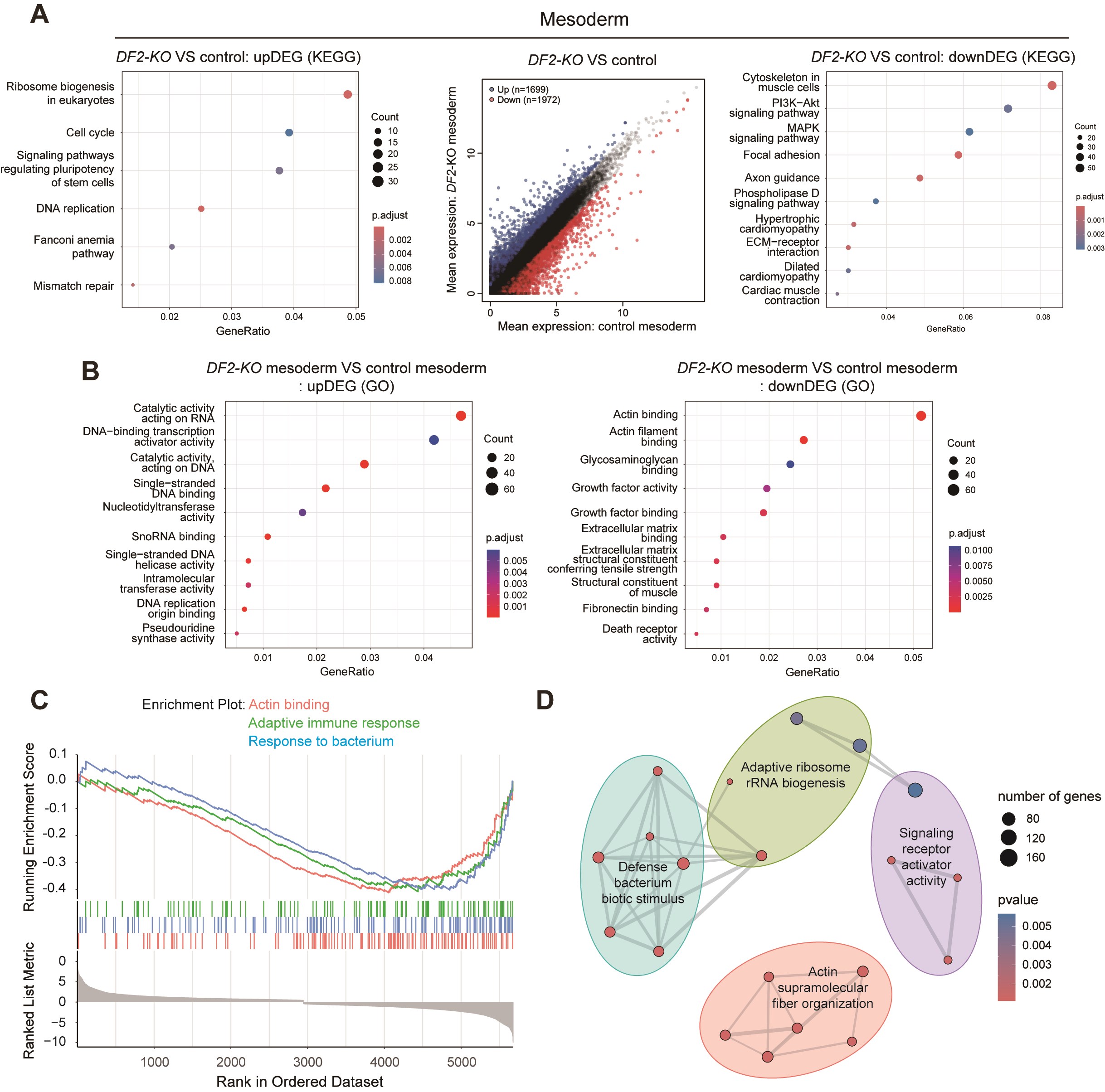

Supplement: sxaf032_suppl_Supplementary_Figure_S5 [file sxaf032_suppl_supplementary_figure_s5.jpeg]

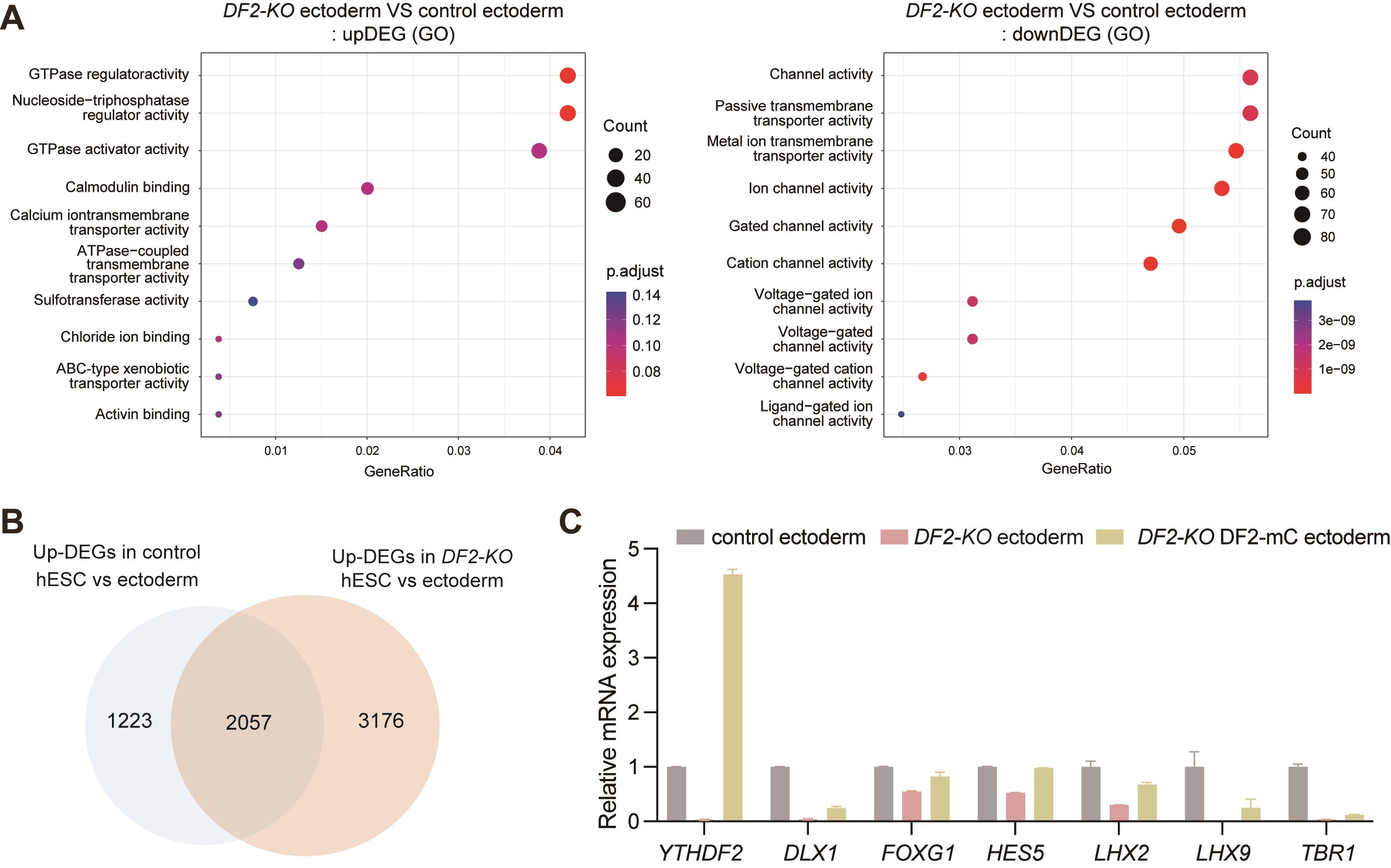

Supplement: sxaf032_suppl_Supplementary_Figure_S6 [file sxaf032_suppl_supplementary_figure_s6.jpeg]

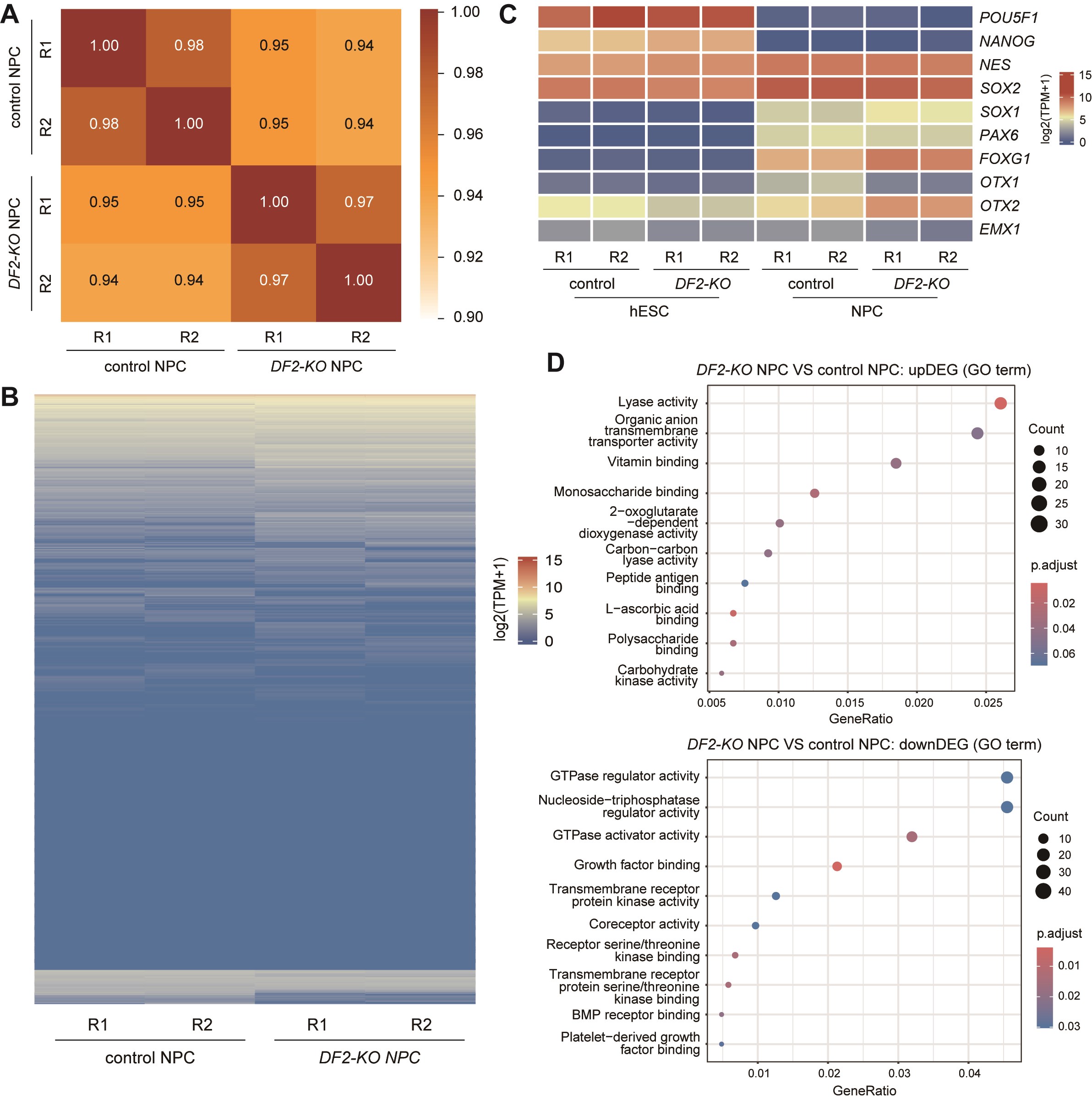

Supplement: sxaf032_suppl_Supplementary_Figure_S7 [file sxaf032_suppl_supplementary_figure_s7.jpeg]

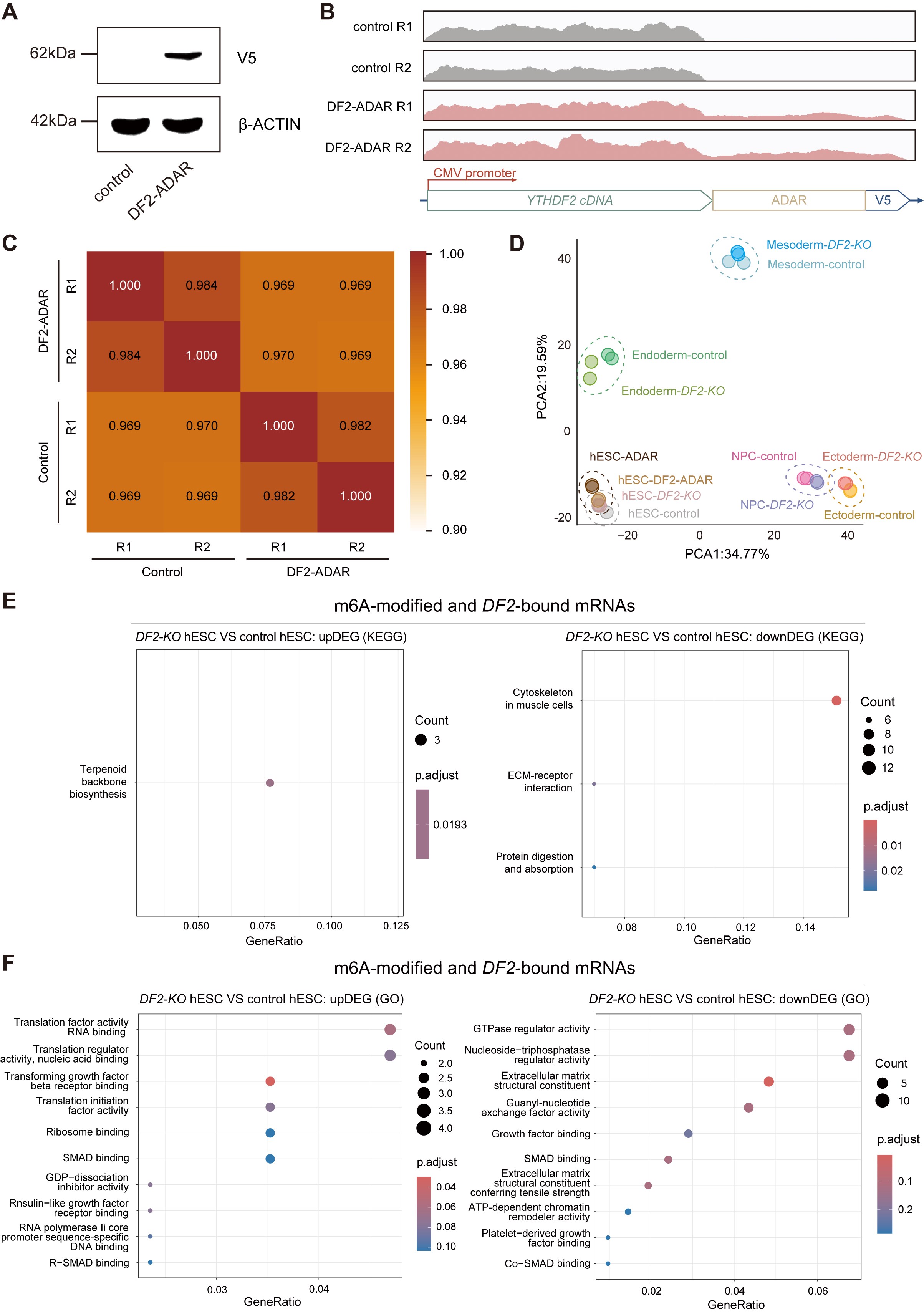

Supplement: sxaf032_suppl_Supplementary_Figure_S8 [file sxaf032_suppl_supplementary_figure_s8.jpeg]

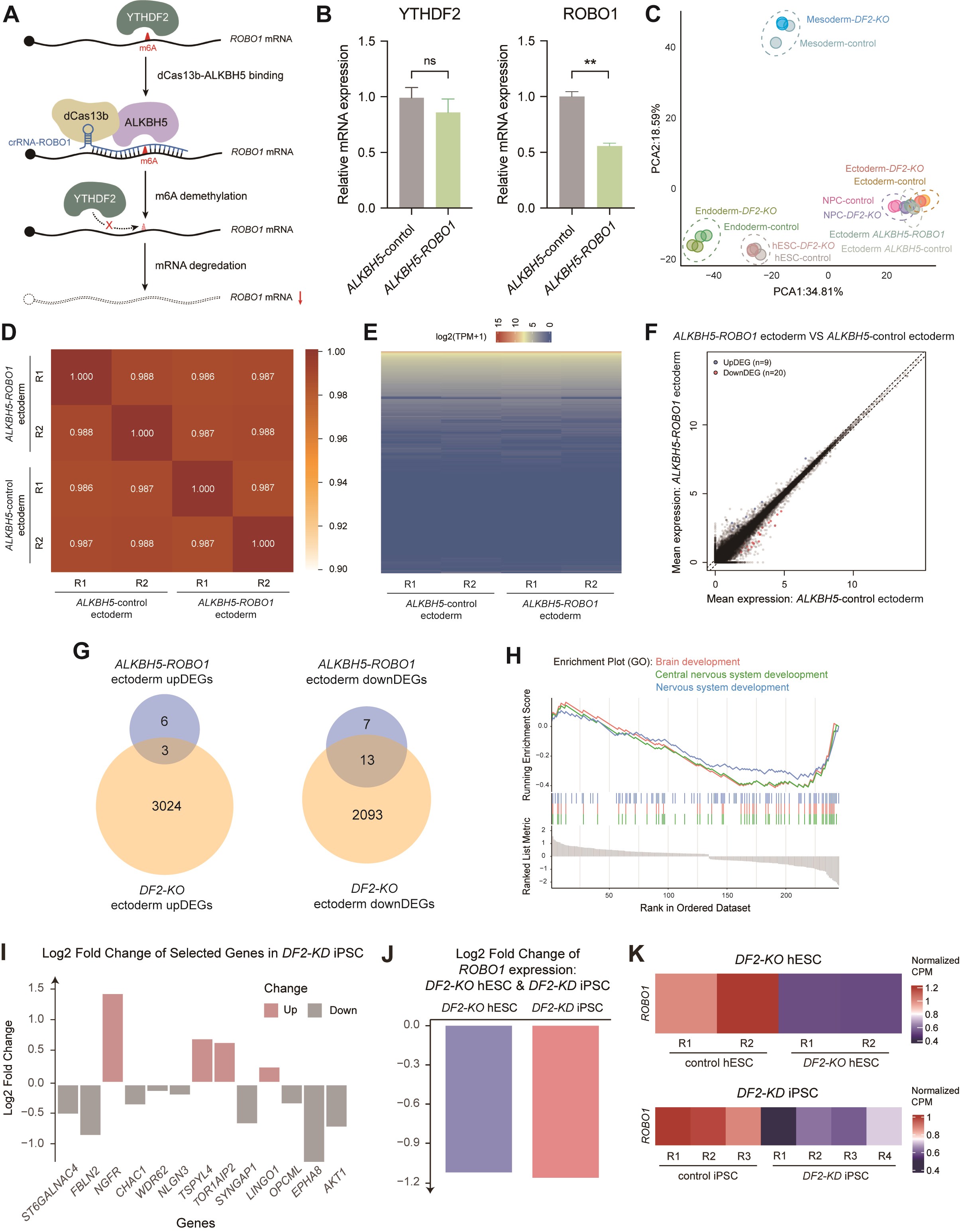

Supplement: sxaf032_suppl_Supplementary_Figure_S9 [file sxaf032_suppl_supplementary_figure_s9.jpeg]
